# Structural basis of human SNAPc recognizing proximal sequence element of snRNA promoter

**DOI:** 10.1038/s41467-022-34639-1

**Published:** 2022-11-11

**Authors:** Jianfeng Sun, Xue Li, Xuben Hou, Sujian Cao, Wenjin Cao, Ye Zhang, Jinyang Song, Manfu Wang, Hao Wang, Xiaodong Yan, Zengpeng Li, Robert G. Roeder, Wei Wang

**Affiliations:** 1 Advanced Medical Research Institute, Cheeloo College of Medicine, Shandong University, Jinan, 250012 China; 2 Department of Biochemistry and Molecular Biology, School of Basic Medical Sciences, Cheeloo College of Medicine, Shandong University, Jinan, 250012 China; 3 Laboratory of Biochemistry and Molecular Biology, The Rockefeller University, New York, 10065 USA; 4 School of Pharmaceutical Sciences, Cheeloo College of Medicine, Shandong University, Jinan, 250012 China; 5 Wuxi Biortus Biosciences Co. Ltd., Jiangyin, 214437 China; 6 Third Institute of Oceanography, Ministry of Natural Resources, Xiamen, 361005 China; 7 Interventional Medicine Department, The Second Hospital, Cheeloo College of Medicine, Shandong University, Jinan, 250033 China

**Keywords:** Cryoelectron microscopy, Transcription factors

## Abstract

In eukaryotes, small nuclear RNAs (snRNAs) function in many fundamental cellular events such as precursor messenger RNA splicing, gene expression regulation, and ribosomal RNA processing. The snRNA activating protein complex (SNAPc) exclusively recognizes the proximal sequence element (PSE) at snRNA promoters and recruits RNA polymerase II or III to initiate transcription. In view that homozygous gene-knockout of SNAPc core subunits causes mouse embryonic lethality, functions of SNAPc are almost housekeeping. But so far, the structural insight into how SNAPc assembles and regulates snRNA transcription initiation remains unclear. Here we present the cryo-electron microscopy structure of the essential part of human SNAPc in complex with human U6-1 PSE at an overall resolution of 3.49 Å. This structure reveals the three-dimensional features of three conserved subunits (N-terminal domain of SNAP190, SNAP50, and SNAP43) and explains how they are assembled into a stable mini-SNAPc in PSE-binding state with a “wrap-around” mode. We identify three important motifs of SNAP50 that are involved in both major groove and minor groove recognition of PSE, in coordination with the Myb domain of SNAP190. Our findings further elaborate human PSE sequence conservation and compatibility for SNAPc recognition, providing a clear framework of snRNA transcription initiation, especially the U6 system.

## Abstract (web summary)

This study presenting the cryo-EM structure of human SNAPc in complex with U6-1 PSE, significantly enforces our understanding of the mechanism of the SNAPc assembly, the PSE-specific recognition by SNAPc and the PIC assembly on U6 snRNA promoter.

## Introduction

Small nuclear RNAs (snRNAs) are a distinct class of highly conserved non-coding RNAs that play a vital role in the survival of eukaryotic cells. Five snRNAs (U1, U2, U4, U5, and U6) constitute the central components of the spliceosome that executes precursor messenger RNA splicing, one of the most fundamental cellular activities. Besides intron removal and exon ligation, snRNAs are also involved in gene transcription regulation (7SK), ribosomal RNA processing (U3), 3’ end formation of histone mRNA (U7) and so on^1,2^. In eukaryotes, snRNAs are tightly regulated to maintain cellular homeostasis in different cell cycle stages or in response to variable cell growth conditions^3^. In the process, snRNA gene transcription is particularly critical, because dysregulations of human snRNA levels are usually accompanied by neurological diseases or tumorigenesis^4–6^.

**Fig. 1:**
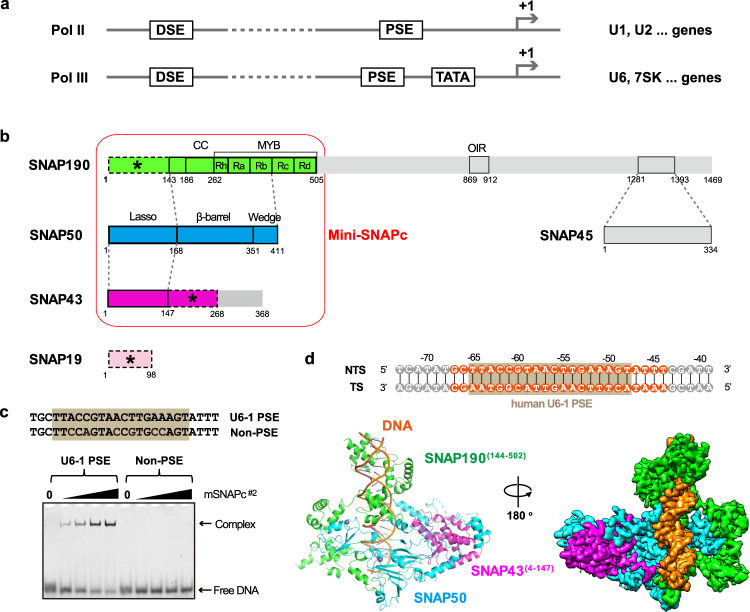
Overall structure of human mini-SNAPc complexed with U6-1 PSE. **a** Schematic diagram of two types of human snRNA promoters to recruit Pol II or Pol III. **b** Five-subunit hSNAPc composition with detailed domain organization. SNAP190^(1-505)^ is shown in green, SNAP50 in cyan, SNAP43^(1-268)^ in magenta, and SNAP19 in pink. The domain–domain interactions are indicated using dash lines. The subunit or fragments not constructed for mSNAPc^#2^, including SNAP45, SNAP190^(506-1469)^, and SNAP43^(269-368)^, are shown in gray. The rod module containing SNAP19, SNAP190^(1-143)^, and SNAP43^(148-268)^ are indicated as dash boxes with asterisks. Mini-SNAPc is highlighted by a red frame. **c** Electrophoretic mobility shift assay (EMSA) of mSNAPc^#2^ with 25 bp human U6-1 PSE and non-PSE sequences. Increasing amounts of proteins (0, 200, 400, 600, 1200 nM) were incubated with 50 nM fluorescently labeled DNA probes. This experiment was repeated independently three times with similar results. **d** Cryo-EM structure of mini-SNAPc in complex with hU6-1 PSE. The 35 bp DNA sequence is numbered at the exact position of hU6-1 promoter, with 24 bp built into structure highlighted in orange and 18 bp PSE boxed in brown. The overall structure of this complex is shown in cartoon and density map. Color coding follows the same color scheme from Fig.1b (same as below unless otherwise specified). NTS non-template strand, TS template strand.

All snRNA genes share a similar promoter architecture of proximal sequence element (PSE), an essential element located at the region of approximately 40–70 base pair (bp) upstream of the transcription start site (TSS)^7^. PSE is recognized by a specific transcription factor, snRNA activating protein complex (SNAPc)^8^, which is also known as PSE-binding transcription factor (PTF)^9^. Another featured common sequence is the distal sequence element (DSE), which is normally found at the position from −250 to −170 bp in many but not all snRNA promoters^3,10,11^. DSE contains a number of protein-binding sites, one of which is an octamer sequence recognized by the transcription activator Oct-1. The Oct-1 POU domain is further involved in a protein-protein interaction with SNAPc to regulate its PSE-binding activity, mediated by a positioned nucleosome^12,13^. In eukaryotes, most snRNAs are transcribed by RNA polymerase II (Pol II), and a small group of snRNAs, such as U6 and 7SK, are synthesized by RNA polymerase III (Pol III)^14^. Despite similar promoter elements, the mechanism of recruited RNA polymerase selectivity can be different in divergent organisms. In vertebrates, a TATA-box located downstream of PSE plays a key role in Pol III-specific snRNA gene transcription, whereas the absence of TATA-box leads to Pol II-specific snRNA gene transcription (Fig. 1a)^15,16^. In *Drosophila melanogaster*, the sequence of PSE is sufficient to determine the selectivity of Pol recruitment^17^. In plants, the key determinant of Pol specificity is the distance between PSE and TATA-box^18^.

SNAPc is a critical transcription factor (TF) in snRNA genes transcription, and its interaction with PSE is the first step to recruit Pol II- or Pol III-specific factors in the assembly of pre-initiation complex (PIC)^11,19^. SNAPc exists in most eukaryotes with three-subunit or five-subunit composition in different species. The vertebrate SNAPc consists of five subunits: SNAP190 (SNAPC4, PTFα), SNAP50 (SNAPC3, PTFβ), SNAP45 (SNAPC2, PTFδ), SNAP43 (SNAPC1, PTFγ), and SNAP19 (SNAPC5) (Fig. 1b). In lower eukaryotes such as *D. melanogaster*, *Caenorhabditis elegans*, and *Arabidopsis thaliana*, SNAPc functions as a three-subunit complex with SNAP190, SNAP50, and SNAP43 homologs. These three subunits are evolutionarily conserved and represent the core subunits of human SNAPc required for PIC assembly on snRNA promoters^19,20^. In human, the largest subunit SNAP190 containing a Myb domain with four and a half MYB repeats and SNAP50 containing two zinc fingers can be UV cross-linked to PSE. However, neither SNAP190 nor SNAP50 is capable of binding to DNA alone. SNAP43 must be involved to form a stable DNA-binding complex^21–23^. The partial complex, termed mini-SNAPc, is composed of N-terminal third part of SNAP190, SNAP50, and SNAP43, and is fully functional for PSE-binding and snRNA transcription^24^. In contrast, SNAP19 and SNAP45 are non-conserved and dispensable for transcription in vitro, but they may be involved in stabilizing complex conformation and regulating transcriptional activity^25,26^. Interestingly, the PSE-binding activity of full-length five-subunit human SNAPc is auto-repressed, while mini-SNAPc can efficiently bind to PSE. This auto-inhibition can be released by the direct interaction between the POU domain of Oct−1 and OIR motif of SNAP190^9,24^, and the recruited TBP (TATA binding protein) can up-regulate SNAPc activity^27^. In human cells, the recruitment of specific polymerase depends on the communications between SNAPc and Pol-specific TFs, as well as the presence or absence of TATA-box at the snRNA promoter. In U6 and 7SK promoters, one type of TFIIIB complex consisting of TBP, Brf2 (TFIIB-related factor 2), and Bdp1 (B double prime 1) binds to TATA-box and coordinates with SNAPc to guide Pol III-specific PIC assembly^28^. In contrast, another transcription factor complex of TBP-TFIIB-TFIIA interacts with SNAPc to initiate Pol II-dependent snRNA transcription at the TATA-less snRNA promoters^29^. Phenotypes for mutated or inactivated SNAPc-related genes are associated with abnormal skeleton morphology, type II diabetes mellitus, and behavioral/neurological disorder, etc (PHAROS database)^30^. Furthermore, gene-knockout of homozygote (SNAP43 or SNAP45) in mouse can lead to embryonic lethality (Mouse Genome Informatics database), which is consistent with the crucial functions of SNAPc in snRNA transcription regulation.

SNAPc was identified two decades ago^8,9^ and in-depth studies (especially in human and fruit flies)^19,20^ make snRNA genes transcription an intriguing system for understanding how RNA polymerase specificity is determined by one common factor under different promoter background. Especially, the U6 promoter has been widely engineered for synthetic RNAs expression in RNAi-mediated knock-down system and CRISPR/Cas9-mediated genome editing system^31,32^. However, the detailed molecular mechanism of how SNAPc assembles and recognizes PSE remains unclear. The lack of direct structural information has hampered our further understanding of snRNA gene transcription regulation. Here we solved the cryo-electron microscopy (cryo-EM) structure of human mini-SNAPc binding to human U6-1 PSE at the overall resolution of 3.49 Å. In this structure, the conserved N-terminal domain (NTD) of SNAP190, SNAP50, and SNAP43 assemble as a stable mini-SNAPc in a “wrap-around” mode. Strikingly, three important motifs of SNAP50 rather than two zinc fingers coordinate with the Myb domain of SNAP190 in PSE-binding. Together with structure-guided in vitro biochemical assays, we preliminarily elucidated the molecular basis of the PSE sequence preference recognized by SNAPc, with five key residues identified. A well-characterized model of PIC assembly on human U6 promoter is proposed to better understand Pol III-dependent snRNA transcription initiation.

## Results

### The extreme N-terminal domain of SNAP190 and the middle domain of SNAP43 are essential for mini-SNAPc stability

Human mini-SNAPc containing SNAP190^(1-505)^, SNAP50, and SNAP43 has been reported to be fully competent for PSE-binding and snRNA transcription in vitro^24^, but SNAP19 may be involved in the assembly of the core complex due to its interaction with N-terminus of SNAP190 and SNAP43 (Fig. 1b)^24^. To understand the composition of SNAPc, we purified four-subunit complex, termed mSNAPc^#1^ (listed in Supplementary Table 1, similarly hereinafter), with mini-SNAPc and SNAP19 co-expressed in insect cells. This complex contains a lower band (Supplementary Fig. 1a) which is identified as the carboxyl terminal degraded SNAP43 by mass spectrometry. Because the CTD (residues 269-368) of SNAP43 has been reported to be dispensable for mini-SNAPc assembly and PSE-binding^33^, a second complex with the CTD of SNAP43 deletion was generated (mSNAPc^#2^) (Supplementary Fig. 1b). As illustrated by the results of gel filtration and electrophoretic mobility shift assay (EMSA), mSNAPc^#2^ acts as a stable complex to specifically recognize human U6-1 PSE rather than non-PSE sequence^23^ in a way similar to mSNAPc^#1^ (Fig. 1c, Supplementary Fig. 1c).

**Fig. 2:**
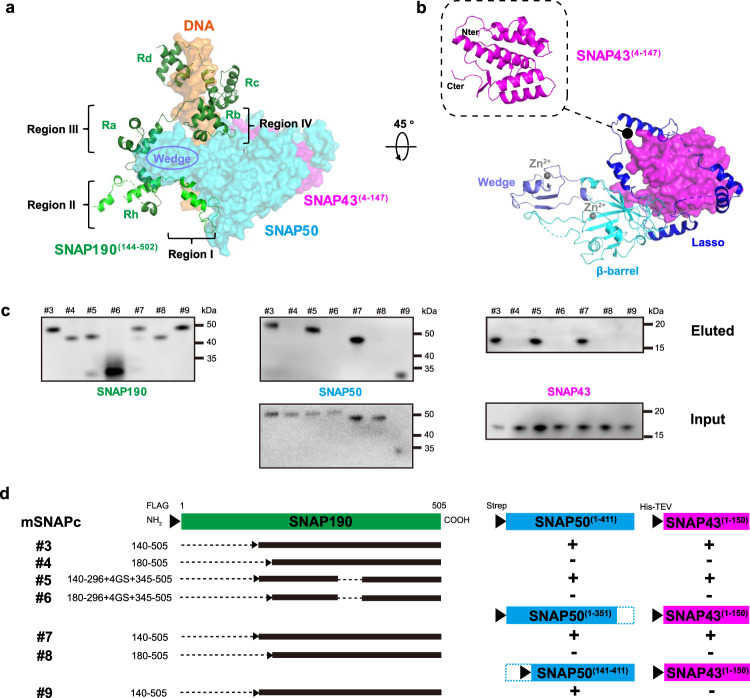
Mini-SNAPc assembles in a “wrap-around” mode. **a** Domain organizations of SNAP190^(144-505)^. Four and a half MYB repeats (Rh, Ra, Rb, Rc, and Rd) are shown in dark green. Four regions of SNAP190 potentially interacting with SNAP50 are indicated. **b** The experimental structures of SNAP50 and SNAP43^(4-147)^. The lasso domain, the β-barrel domain and the Wedge domain are shown in blue, cyan, and light blue, respectively. Two zinc atoms are labeled as gray dots. The NTD of SNAP43 is embedded into an interface formed by the lasso domain of SNAP50. **c**, **d** Domain–domain interaction analysis within mini-SNAPc using anti-FLAG affinity chromatograph. Western blots showed that Region I is the main segment of SNAP190 interacting with SNAP50, and SNAP50 relies on the Lasso domain to grab the NTD of SNAP43. Detailed in Method Section. This experiment was repeated independently three times with similar results.

Finally, we solved the cryo-EM structure of mSNAPc^#2^ in complex with 35 bp human U6-1 PSE-containing sequence at the overall resolution of 3.49 Å (Fig. 1d, Supplementary Figs. 2, 3). The clear density fits perfectly with double-stranded DNA (dsDNA), which covers 18 bp human U6-1 PSE conserved sequence (ranked from position −65 to −48 relative to TSS) (Fig. 1d, Supplementary Figs. 3e, f). With the exception of some disordered regions, the atomic model of SNAP190^(144-502)^, SNAP43^(4-147)^, and SNAP50^(28-411)^ are built well de novo into the other visible densities (Fig. 1d, Supplementary Fig. 3e). The modeled part of mSNAPc^#2^ happens to be the conserved regions of mini-SNAPc that were previously compared with fruit fly three-subunit SNAPc^34^. The densities of the extreme N-terminal domain (residues 1-143) of SNAP190, the middle domain (residues 148-268) of SNAP43, and subunit SNAP19 are missing in our structure. It is implicated that they could be flexible and do not participate in DNA-binding. To verify our model, we tried to purify the complex corresponding to the built model (mSNAPc^#3^). As shown in western blot (Fig. 2c, d), SNAP190^(140-505)^ with N-terminal FLAG tag was co-purified with SNAP43^(1-150)^ and SNAP50 after anti-FLAG affinity chromatography. However, this complex is so unstable that it rapidly dissociates or degrades in vitro. In contrast, we were able to get one stable sub-complex of SNAP190^(1-143)^, SNAP43^(148-268)^, and SNAP19 (mSNAPc^#11^), without DNA-binding activity detected (Supplementary Figs. 1d, e). This result is consistent with previous studies that these fragments can interact with each other, whose long α-helices might fold into a coiled-coil conformation^35^. More evocatively, we designate mSNAPc^#11^ as the rod module. Considering that deletion of non-conserved subunit SNAP19 does not affect mini-SNAPc assembly and DNA-binding^24^, the extreme NTD of SNAP190 and the middle domain of SNAP43 are crucial for the stability of mini-SNAPc. In view of the missing rod module information, our structure actually deciphers an architecture of mini-SNAPc (the term is adopted to describe SNAPc structural part hereinafter) in complex with U6 PSE.

### The assembly of human mini-SNAPc complex in the “wrap-around” mode

For the mini-SNAPc assembly, there exist multiple interfaces of SNAP50-SNAP190^(144-502)^ and SNAP50-SNAP43^(4-147)^, but no contact between SNAP190^(144-502)^ and SNAP43^(4-147)^. Briefly, in DNA-binding state, SNAP50 constitutes the scaffold of mini-SNAPc architecture and it is surrounded by SNAP190^(144-502)^ and SNAP43^(4-147)^. The subunit of SNAP50 does not have any known homologous structure, and we divided it into three domains, namely N-terminal Lasso domain, middle imperfect β-barrel domain mainly composed of one α-helix and eight β-sheets, and C-terminal Wedge domain (Fig. 2b). Two zinc fingers are located in the β-barrel domain and Wedge domain, respectively. However, these zinc fingers function as the structural component rather than PSE-recognition motifs, as illustrated in the structure (Supplementary Fig. 5f). SNAP50 mainly depends on β-barrel domain and Wedge domain to interact with SNAP190, and Lasso domain and β-barrel domain to wrap around SNAP43.

The most remarkable characteristics of SNAP190 is a Myb domain that extends from residues 263 to 505, with four and a half MYB repeats (Rh, Ra, Rb, Rc, and Rd). Four regular MYB-repeats have all three short α-helices (α1, α2 and α3), whereas Rh only contains α2 and α3. As shown in the structure, Rb, Rc and Rd bind tightly to the major groove of dsDNA, whereas Rh and Ra are not engaged in DNA-binding. Except Rc and Rd, many residues of SNAP190^(144-502)^ participate in an elaborate network of interactions with SNAP50, which are divided into four regions (Fig. 2a). Region I (residues 144-179) is mainly docked in β-barrel domain of SNAP50. Region II is comprised of Rh and CC (coiled-coil) domain. The latter is formed by two long α-helices via intrinsic hydrophobic interactions, whose distal part is not visible in the map due to flexibility. Region III and IV are referred to as Ra and Rb, respectively. These four regions enclose the Wedge domain of SNAP50 (Fig. 2a). Based on structural analyses, SNAP190 probably binds to SNAP50 via Region I and Region III, because both regions contain a series of hydrophobic residues inserting into the concave surface of SNAP50. On the contrary, fewer residues of Region II and Region IV are in contact with SNAP50 (Supplementary Fig. 4). To verify our structural information on the interaction between SNAP190 and SNAP50, we performed co-expression and anti-FLAG affinity chromatography experiments of several SNAPc constructs with key domain deletions (Fig. 2c, d). As aforementioned, full-length SNAP50 and SNAP43^(1-150)^ were co-purified with N-terminal FLAG-tagged SNAP190^(140-505)^. When Region I was deleted, western blot results showed that there was only SNAP190^(180-505)^ band, but in the absence of SNAP43^(1-150)^ and SNAP50 bands. However, substitution of Region III (Ra) with 4GS-linker on SNAP190 does not eliminate its interactions with SNAP50 and SNAP43^(1-150)^. This result suggests that Region I rather than Region III plays a key role in mediating SNAP190 and SNAP50 interaction. Even when we further deleted the Wedge domain of SNAP50, SNAP190^(140-505)^ was still eluted with SNAP50^(1-351)^ and SNAP43^(1-150)^. Hence, the phenomenon of the Wedge domain of SNAP50 enclosed by four regions of SNAP190 could be due to one specific conformation of mini-SNAPc in DNA-binding state.

The solved structural part of SNAP43 contains two segments (Fig. 2b). Residues 1-138 form a global, compacted domain with seven α-helices and two short β-sheets. The second segment is the loop (residues 139-147), which spans SNAP50 and extends the middle domain of SNAP43 to interact with the extreme NTD domain of SNAP190 and SNAP19. Residues 28-66 of SNAP50 constitute an “Anchor motif” to mediate the interaction between the β-barrel domain of SNAP50 and the NTD of SNAP43, relying on extensive van der Waals forces and several hydrogen bonds (Supplementary Fig. 5a–c). Furthermore, it is fastened to the β-barrel domain to function as a “knot”, which allows other NTD part (residues 67-168) of SNAP50 to encircle SNAP43 like a lasso. Within the lasso, two fragments (residues 77-109 and 117-143) are in close contact with SNAP43 (Supplementary Fig. 5d, e). We also performed the anti-FLAG affinity chromatography, in which the Lasso domain of SNAP50 is deleted. The FLAG-tagged SNAP190^(140-505)^ eluted with SNAP50^(141-411)^ but not SNAP43^(1-150)^ (Fig. 2c, d). This result clearly demonstrates that the Lasso domain of SNAP50 is vital for interacting with the NTD of SNAP43.

### Structural basis of mini-SNAPc binding to human U6-1 PSE

**Fig. 3:**
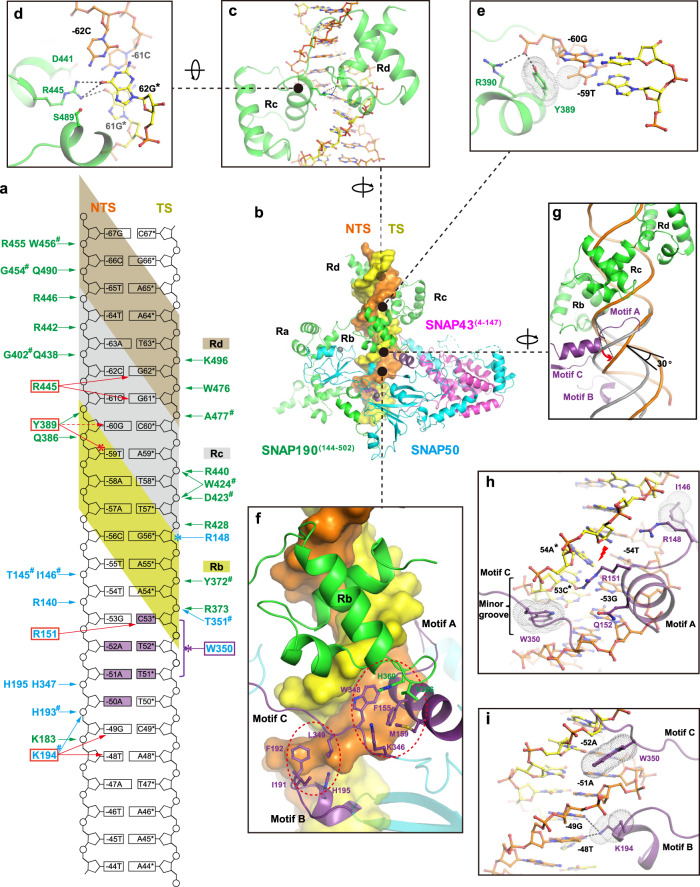
Structural insight into PSE-recognition by mini-SNAPc. **a** Schematic representation of 24 bp hU6-1 PSE interacting with the corresponding residues of SNAPc. The hydrogen bonds and hydrophobic interactions are shown with arrows and clubs, respectively. Those residues using the main chain to interact with DNA are labeled with a hash (#). Residues guiding specific base-interaction via hydrogen bonds are boxed in red, with one exception of W350 relying on hydrophobic interaction colored in purple. **b** Structural representation of PSE-recognition by mini-SNAPc. The DNA duplex is shown as surface, in which NTS and TS are colored in orange and yellow, respectively. **c**, **d** Close-up views of the Rc and Rd binding to PSE duplex. R445 of Rc domain forms hydrogen bonds with GC pairs at positions -61 and -62. D441 and S489 could help conformational stability of the side chain of R445. **e** Y389 of Rb is fixed in one specific conformation with the help of -60G and -59T. **f** SNAP50 recognizes PSE through three Motifs (A, B, and C colored in purple cartoon). Two hydrophobic cores are highlighted by red dashed circles. **g** The short helix of Motif A of SNAP50 inserts into the minor groove of PSE duplex by expanding ~4 Å (red arrow). As a result, the downstream of PSE is distorted by 30°. The regular B-form dsDNA is shown in gray cartoon. **h** The first hydrophobic core consists of Motif A and C, which insert into the minor groove. W350 is in charge of nucleotides-recognition through van der Waals forces. R151 of Motif A recognizes GC pair at position -53 by forming a hydrogen bond. As a result of the widening of minor groove, the hydrogen bonds of TA pair at position -54 are broken (red lightning). **i** The second hydrophobic core is composed of Motif B and C. The NTS of PSE is sandwiched by W350 and K194 in π-π stacking mode. K194 of Motif C also forms hydrogen bonds with -49G and -48T. The sticks of Y389, -59T, W350 and K194 participating in hydrophobic interactions are covered by dot densities.

One striking feature of the overall structure is that mini-SNAPc wraps around the PSE duplex halfway, which is adopted as B-form DNA. Twenty-four consecutive Watson-Crick base pairs were built well into the density, which completely cover the 18 bp human U6-1 PSE sequence (Fig. 1d, Supplementary Figs. 3e, f). The PSE-binding is executed by SNAP190 and SNAP50 subunits. In SNAP190, only Myb domain participates in DNA-binding, which is in accordance with previous studies^23^. The MYB-containing proteins are found as eukaryotic TFs and recognize promoters of target genes in a sequence-specific manner. The MYB superfamily is divided into three main types: single-repeat, two-repeat (R2R3) and three-repeat (R1R2R3) MYB proteins, with one notable exception of hSNAP190-like protein containing 4.5 MYB repeats^36^. In most cases of three-repeat proteins, R1 is dispensable for the specific binding of target sequences, while R2 and R3 cooperatively bind to the DNA major groove. This mode is similar to two-repeat MYB proteins. Unexpectedly, in SNAP190, three MYB repeats of Rb, Rc, and Rd dock into the major groove, whereas Rh and Ra mainly take part in SNAPc assembly (Fig. 3b). A large number of interactions with the sugar-phosphate backbone of DNA occur between three MYB repeats and 5’ half of PSE (Fig. 3a, Supplementary Fig. 6). By contrast, the Myb domain makes fewer base-specific DNA contacts, with only two residues with clear side chain density in cryo-EM map observed to be involved. The first one is R445 of Rc domain, which forms hydrogen bonds with the purine groups of CG pairs at positions −62 and −61. D441 of Rc and S489 of Rd, located on the flanks of R445, might play a role in stabilizing side chain conformation of R445 (Fig. 3c, d). However, the observed interactions here should be limited because of the missing information of water-mediated hydrogen bonds at the current resolution. For instance, the N7 atoms of guanines from both CG pairs here might form a stronger H-bond network with R445 or/and S489 mediated by waters (Supplementary Fig. 12b). Y389 of Rb is the second residue guiding base-specific recognition. The side-chain of Y389 adopts a specific conformation mainly fixed by two efforts: the methyl group of −59T forms hydrophobic interaction with the aromatic ring of Y389, and the OH group of Y389 forms one hydrogen bond with phosphate group of −60G (Fig. 3e). Besides, a water could mediate hydrogen bonds between N7 atom of −60G and OH group of Y389 to further stablize Y389 conformation (Supplementary Fig. 12c).

SNAP50 recognizes the 3’ half of PSE mainly by interacting with the minor groove (Fig. 3b). The DNA-binding part encompasses three motifs. Motif A consists of a loop (residues 144-150) and the helix (residues 151-160) of the Lasso domain. This motif cannot interact with the NTD of SNAP43. Motif B is composed of a loop (residues 188-192) and a short helix (residues 193-198). Motif C is the loop (residues 346-353) that connects the β-barrel domain and the Wedge domain. These motifs are organized spatially to form two hydrophobic cores (Fig. 3f). The first hydrophobic core is mainly formed by F155 of Motif A, W348 of Motif C, and H369 of SNAP190. It provides the direct structural evidence that SNAP190 and SNAP50 work in concert to bind DNA effectively. In this hydrophobic core, one key arginine of Motif A is responsible for base-specific interaction: R151 can form hydrogen bonds with CG pair at position −53. Due to the presence of R151 and Q152 of α-helix (residues 151-160), the minor groove is widened by ~4 Å compared to the regular B-form dsDNA (Fig. 3g). Furthermore, the hydrogen bonds of AT pair at position −54 are broken, and the downstream DNA is bent by 30 degrees (Fig. 3g, h). The side chain of another arginine R148 also inserts into the minor groove, but its poor density shows that it is highly flexible and likely interacts with the backbone of DNA. On both sides of the hydrophobic core, the side chains of Motif A I146 and Motif C W350 insert into the narrow minor groove like two crab claws via van der Waals force (Fig. 3h). The second hydrophobic core mainly comprises I191-F192 of Motif B and L349 of Motif C (Fig. 3f). The short helix (residues 193-198) is docked in the major groove, in which K194 can interact with “GpT” at positions −49 and −48. Therefore, the non-template strand (NTS) of PSE is sandwiched between W350 of Motif C and K194 of Motif B (Fig. 3i). Besides these residues specially “sensing” the base of PSE, many other residues of SNAP50 are involved in extensive hydrogen bonds with the phosphate backbone of DNA (Fig. 3a, Supplementary Fig. 6). These non-specific protein-DNA interactions further strengthen the stability of SNAPc-PSE complex.

**Fig. 4:**
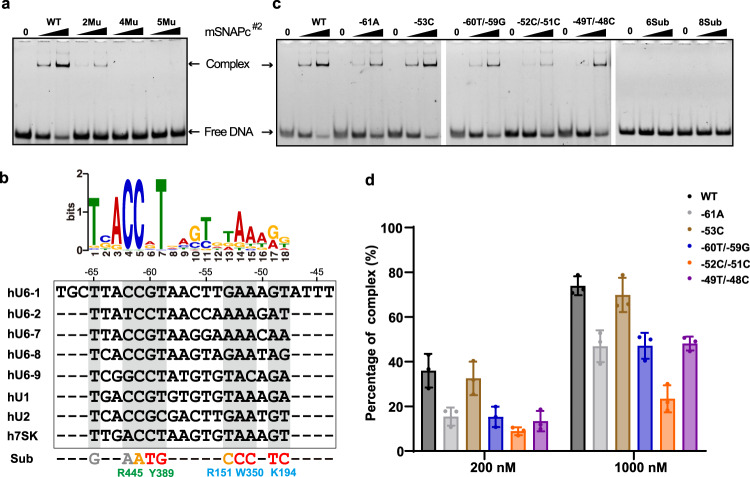
PSE-recognition relies on synergistic action of multiple residues of SNAPc. **a** The binding activities of WT, 2Mu, 4Mu, 5Mu mSNAPc^#2^ with U6-1 PSE were analyzed by EMSA. For each experiment, 200 and 1000 nM proteins (WT or mutant) were mixed with 50 nM fluorescently labeled 25 bp DNA probes. This experiment was repeated independently three times with similar results. **b** The top panel showed a LOGO profile of human PSE based on sequence alignments of known human PSEs. The PSE sequences of human U6-1, U6-2, U6-7, U6-8, U6-9, U1, U2, and 7SK are in alignment. Residues of SNAPc involved in specific recognition of corresponding nucleotides are shown on the bottom. The substituted nucleotides designed to test binding capacity between SNAPc and “mutated” PSE are labeled in different colors: six-point substituted PSE (6Sub) contains red bases, and eight-point substituted PSE (8Sub) contains red and orange bases. **c** Representative EMSA of mSNAPc^#2^ and seven substituted PSEs, designed around five key residues of SNAPc (namely –61A, –53C, –60T/–59G, –52C/–51C, –49T/–48C, 6Sub, and 8Sub). **d** Quantitative analysis of above-mentioned EMSA. Data are mean ± SEM (*n* = 3). The percentage of arrested DNA-protein complex (*Y* axis) was calculated under two different concentrations of mSNAPc^#2^ (200 and 1000 nM, *X* axis) incubated with 50 nM fluorescently labeled DNAs. Source data are provided as a Source Data file.

Sequence alignments shows that most residues involved in DNA-binding are highly conserved among SNAP190 and SNAP50 homologs (Supplementary Figs. 7, 8). To understand the mechanism of how SNAPc specifically binds to PSE over other promoter elements, we introduced a series of residue mutations into the recombinant mSNAPc^#2^, with two residues of SNAP190 (Y389 and R445) and four residues of SNAP50 (R148, R151, K194 and W350) replaced by alanine. Five residues therein should take charge of base-specific recognition on the basis of aforementioned structural analysis. Although the side chain of R148 does not point toward the bases of PSE, its location inside minor groove and poor density shows that this flexible residue probably still participates in nucleotide-specific interaction. We first purified single-point mutants of mSNAPc^#2^ to test the differences in DNA-binding affinity using Surface Plasmon Resonance (SPR) (Table 1, Supplementary Fig. 10). Proteins were covalently coupled to the chip, and 25 bp human U6-1 PSE at HPLC-purified level was injected into the microfluidic channel. The wild type (WT) complex with 25 bp PSE yielded a K_D_ of 0.289 μM. The curve type also indicates that DNA can dissociate from SNAPc quickly. Among six single-mutants, Y389A of SNAP190 and R151A of SNAP50 are the more prominent residues in decreasing DNA-binding affinity, with ~4-fold reduction. The single-point Ala-substitution on W350 of SNAP50 or R445 of SNAP190 resulted in 2~3-fold reduction. The other two mutations (R148A or K194A of SNAP50) had less impact on DNA-binding affinity. Taken together, these single-point mutations had no significant effect on PSE-binding activity of SNAPc. The EMSA experiments of 25 bp PSE duplex with these single-point mutants further confirmed the conclusion from SPR (Supplementary Fig. 11b). Subsequently, on the basis of K_D_-weakening trend of single-point mutants, two-point (Y389A of SNAP190 and R151A of SNAP50), four-point (Y389A, R445A of SNAP190, and R151A, W350A of SNAP50), and five-point (all except for non-specific R148 of SNAP50) mutated proteins were generated. The SPR result showed that K_D_ value of four-point and five-point mutants binding to PSE dramatically decreased to 8.910 and 48.950 μM, respectively (Table 1, Supplementary Fig. 10). In EMSA, no PSE-binding band was observed for the four-point and five-point mutants (Fig. 4a), because such low PSE-binding affinity is not sufficient to maintain a stable DNA-protein complex in native gel. Therefore, the biochemical assays with multiple-residue mutants clearly demonstrated that Y389 and R445 of SNAP190, and R151, K194 and W350 of SNAP50 play important roles in guiding PSE-specific binding, which is highly consistent with our structural model.

**Table 1:** Kinetics and affinity constants for wild-type and mutated mSNAPc^#2^ binding to 25 bp hU6-1 PSE duplex revealed by SPR

Protein	Association rate (ka, *M*^*−1*^ *s*^*−1*^)	Dissociation rate (kd, *s*^*−1*^)	Binding affinity (KD, *M*)
WT	1.082 × 10^5^	0.03125	2.889 × 10^‒7^
SNAP190-Y389A	9.174 × 10^4^	0.09955	1.085 × 10^‒6^
SNAP190-R445A	6.237 × 10^4^	0.03758	6.026 × 10^‒7^
SNAP50-R148A	7.114 × 10^4^	0.03970	5.581 × 10^‒7^
SNAP50-R151A	2.693 × 10^4^	0.03091	1.148 × 10^‒6^
SNAP50-K194A	1.048 × 10^5^	0.05374	5.127 × 10^‒7^
SNAP50-W350A	5.097 × 10^4^	0.04522	8.871 × 10^‒7^
2Mu	1.838 × 10^4^	0.02978	1.620 × 10^‒6^
4Mu	4.945 × 10^4^	0.4406	8.910 × 10^‒6^
5Mu	3.521 × 10^3^	0.01724	4.895 × 10^‒5^

### The conservation and compatibility of versatile PSE sequences based on mini-SNAPc/U6-1 model

There are many U6 loci dispersed throughout the genome. Of them, five U6 genes (U6-1, U6-2, U6-7, U6-8 and U6-9) have been shown to be active, which all contain complete DSE, PSE, and TATA elements^37^. Besides U6 PSE, SNAPc complex can also recognize other PSE sequences, such as U1, U2, and 7SK genes^3^. These PSE sequences are highly conserved, despite a low degree of variability exists (Fig. 4b). We further summarized the reported human snRNA promoters^14^ to generate a LOGO of PSE (Fig. 4b). Based on information of the LOGO and our mini-SNAPc/U6-1 model, ten single nucleotide substitutions were reverse designed into human U6-1 PSE duplex. EMSA and structural modeling were combined to preliminarily analyze the PSE conservation and compatibility recognized by SNAPc (Supplementary Figs. 12, 13a). In EMSA experiments, these 25 bp PSE variants were quantitatively analyzed with mSNAPc^#2^. These nucleotide replacements can weaken SNAPc-binding activity at various degrees, which is consistent with the following structural analysis. (1) At position –65, the replacement of T by G only slightly affected SNAPc-binding. Correspondently, no residues of Rd in SNAP190 were observed to guide base-specific recognition. (2) At positions –62 and –61, the side chain of R445 mainly points toward the guanine parts of two CG pairs. Thus, by itself or with the help of waters, R445 could form a H-bond network with O6 and N7 atoms of guanines. When the “AT” pair was introduced, the substitution at position –62 or –61 crippled the interaction with SNAPc. The methyl group of thymine probably breaks the original H-bond network (Supplementary Fig. 12b). (3) At positions –60 and –59, the “TpG” was introduced to replace “GpT”. The structural modeling showed that the methyl group of –60T could clash with the OH group of Y389, and –59G could attenuate hydrophobic interaction with the aromatic ring of Y389 (Supplementary Fig. 12c). As expected, both substitutions moderately impaired SNAPc-binding. (4) At position –53G, the less conserved C was introduced. Although R151 of SNAP50 was detected to be one of the most important residues to guide base-specific interaction by mutated protein assays above, the nucleotide substitution at position -53 only slightly weakened SNAPc-binding. It is hypothesized that the hydrogen bond with R151 is still maintained, despite different base pair replacement (Supplementary Fig. 12d). Two tryptophans (W348 and W350) surrounding R151 block potential water-mediated hydrogen bonds, which is in sharp contrast to R445. (5) At positions –52 and –51, the “GpG” substitution of “ApA” sensed by W350 of SNAP50 dramatically debilitated the interaction with SNAPc. Structural modeling clearly revealed that the portion of GC pair towards the minor groove is more hydrophilic than AT pair (the N2 atoms of guanines are close to W350). Thus, these substitutions can hinder W350 from inserting into the minor groove via hydrophobic interaction (Supplementary Fig. 12e). (6) At positions –49 and –48, the “T” and “C” were introduced, respectively. EMSA results showed that the methyl group of -49T could clash with the side chain of K194 so as to disrupt the original hydrogen bonds more severely than -48C (Supplementary Fig. 12f).

We further compared SNAPc-binding capabilities of single- or double-nucleotide substituted PSEs, designed around the five key residues. The weaken tendency of SNAPc interaction is similar to the single-nucleotide EMSA assay above (Fig. 4c, d), but no base-change around single residue can abolish SNAPc-binding ability. This could be the basis of compatibility of different PSE sequences bound by the same TF, SNAPc. This mechanism guarantees that SNAPc can still recognize PSE, even if one conserved nucleotide is mutated in the genome. Furthermore, EMSA results reflected that R151 of SNAP50 is probably assigned differently from other residues in PSE-binding. The role of R151 is likely to be docking onto the minor groove of PSE rather than base-specific recognition. The docking is accompanied by distorting the PSE orientation to interact with SNAPc more favorably (Fig. 3g). Finally, multiple point substitutions (including six or eight aforementioned nucleotides) were introduced into the same duplex, and both modified PSEs could not form retardant bands of protein-DNA complex in native gels (Fig. 4c). This result confirmed that the recognition of PSE rather than other DNA elements is dependent on the synergistic action of multiple residues of mini-SNAPc.

## Discussion

**Fig. 5:**
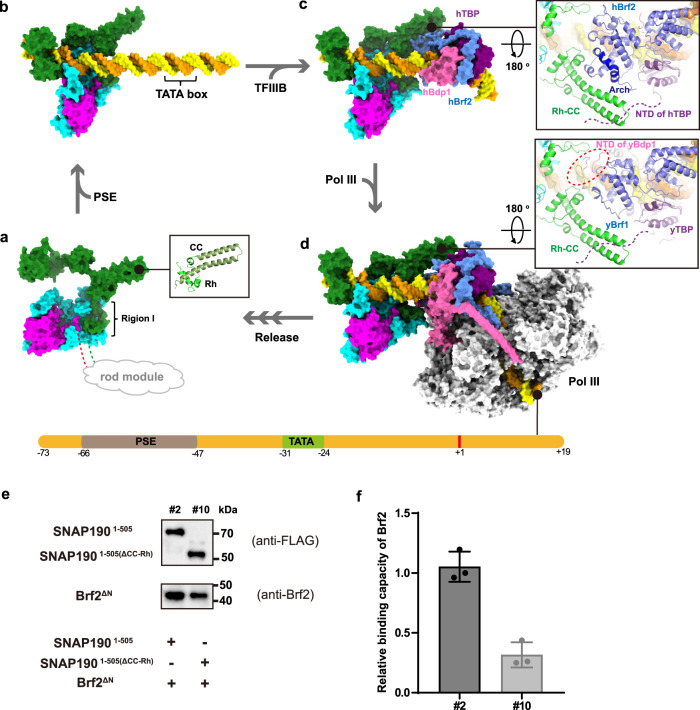
The process of PIC assembly at human U6 promoter. **a** Model of the core region of human SNAPc in PSE-free state. Region I of SNAP190 mediates the interaction with SNAP50, while other regions are flexible, of which the extreme NTD interacts with the middle domain of SNAP43 and SNAP19 to form the rod module. The Rh segment and predicted CC domain of SNAP190 form a complete MYB repeat. **b** The structure of mini-SNAPc recognizing hU6 PSE (this study). **c** A model of hU6 promoter bound by mini-SNAPc and TFIIIB^Brf2^, in which the latter specifically recognizes TATA box (PDB 5N9G). Rh-CC domain of SNAP190 could form an interface with specific SNAPc-interacting Arch motif of hBrf2 (right panel). **d** An integrated human PIC model assembled at U6 promoter using a yeast Pol III-TFIIIB^Brf1^ structure (PDB 6EU0) as reference. The model shows that the NTD of Bdp1 might participate in communication with mini-SNAPc. **e** Pull-down assay to compare Brf2-binding capacity between mSNAPc^#2^ and mSNAPc^#10^. The latter is a construct of mSNAPc^#2^ with CC-Rh domain deleted. **f** The quantitative analysis based on three independent western blot experiments. Data are mean ± SEM (*n* = 3). Detailed in Method Section. Source data are provided as a Source Data file.

PSE is a significant element of snRNA gene promoters, which is specifically recognized by SNAPc. This event is the first step of PIC assembly to trigger the related gene transcription. In this study, we reported the 3.49 Å cryo-EM structure of human mini-SNAPc complex binding to U6-1 PSE. Combining with biochemical and biophysical analysis, our structure answered two fundamental questions in the field of snRNA gene transcription. (1) How do three conserved subunits including the NTD of SNAP190, SNAP50, and SNAP43 (Supplementary Figs. 7–9), assemble into mini-SNAPc with high PSE-binding and transcription activities? Primarily, SNAP50 constitutes the skeleton of the entire complex. On one hand, N-terminal Lasso domain of SNAP50 wraps around the NTD of SNAP43 tightly. On the other hand, the extensive van der Waals forces between the β-barrel domain of SNAP50 and region I of SNAP190 constitute the main interface of these two subunits. In DNA-free state, other regions of SNAP190 probably do not come in contact with SNAP50 (Fig. 5a). After recognizing PSE, regions II-IV of SNAP190 encircles the C-terminal Wedge domain of SNAP50 to form a more stable PSE-binding complex (Fig. 5b). In addition, the extreme NTD of SNAP190, the middle domain of SNAP43, and SNAP19 form a rod module. This module has no DNA-binding activity but is essential for maintaining mini-SNAPc stability. (2) How does mini-SNAPc specifically recognize the PSE sequence rather than other DNA elements? In agreement with previous studies, our structure clearly shows that SNAP190 and SNAP50 work in concert to bind PSE. The high local resolution of PSE-binding regions enable us to provide molecular details of mini-SNAPc interacting with PSE. SNAP190 mainly relies on three MYB-repeats (Rb, Rc, and Rd) of the Myb domain to dock into the 5’-half major groove of PSE. Unexpectedly, only two residues are responsible for nucleotide-specific recognition, while many basic amino acids are involved in binding to the phosphate backbone. MYB repeat is widely adopted by many TFs to recognize specific promoter sequence^36^. It is rarely seen that only a few residues of three MYB repeats are involved in nucleotide-specific recognition. Protein sequence comparison was performed to selectively analyze these residues pointing toward dsDNA major groove (Supplementary Fig. 7). Most of them are highly conserved, which means the SNAP190 per se is insufficient for PSE-specific recognition and SNAP50 must be involved. Three short motifs (Motif A–C) of SNAP50 were identified to be essential for PSE-binding, rather than two zinc fingers. Motif A and C are inserted into the minor groove and Motif B is docked on the 3’-half major groove of PSE. Combined with three MYB repeats of SNAP190, mini-SNAPc binds tightly to PSE in a sandwich mode of “major–minor-major” groove (Fig. 3b). Notably, the Rb of SNAP190 is associated with Motif A and Motif C of SNAP50 to spatially form a hydrophobic core, which directly reflects the synergistic effect to recognize PSE by two subunits. A short α-helix of Motif A inserts into the minor groove of PSE expanding its width by ~4 Å. As a result, the PSE duplex is distorted by ~30^o^ compared with regular B-form dsDNA (Fig. 3g). The bent-conformation of PSE in complex with SNAPc could be more accessible for other TFs or Pol in the PIC assembly. Within these PSE-binding modules, Y389 and R445 of SNAP190, together with R151, K194 and W350 of SNAP50, have been identified as sensors for PSE recognition by bilateral protein mutation and nucleotide substitution experiments.

We also compared our experimental structures with the predicted models generated by AlphaFold server^38^ (Supplementary Fig. 14). Three rigid domains including the β-barrel and Wedge domains of SNAP50, and the NTD of SNAP43, matched quite well. The conformations of the Lasso domain of SNAP50 and the NTD of SNAP190 are obviously divergent, while the subtle domains, such as several MYB repeats still fit perfectly. Structural comparison results showed that the compact domains without long flexible loops can be accurately predicted by AlphaFold. Hence, we filled in the missing part of SNA190 CC-Rh domain, which is well-adapted to our solved structure. One of the two long helices happens to form a complete MYB repeat together with Rh (Fig. 5a).

SNAPc-dependent promoters fall into two categories: Pol II snRNA promoters (U1, U2, etc.) and type 3 pol III promoters (U6, 7SK, RPPH1, etc.)^3,19^. A common feature of these genes is that the regulatory elements are gene-external (generally upstream). In human, the determinant that specifies Pol II or Pol III recruitment is the absence or presence of a TATA box located downstream of the PSE. When TATA box is present, SNAPc mainly recruits Brf2-type TFIIIB complex, a principal transcription initiation factor for Pol III-dependent snRNA promoters. This complex consists of three subunits: TBP, Brf2, and Bdp1. TBP is a general TF shared by all Pols regardless of the absence or presence of TATA box. Brf2 is a non-canonical Pol III-dependent TFIIB-like TF, which replaces Brf1 in a small set of Pol III promoters, such as U6 snRNA and the selenocysteine tRNA^39^. Bdp1 is unique to the Pol III transcription system and is essential for PIC assembly and DNA strand separation of PIC from a closed to open state^40^. A purified transcription system containing mini-SNAPc, TFIIIB^Brf2^, and Pol III can trigger transcription initiation of U6 gene in vitro^41^. Human TBP, Brf2, and Bdp1 have been reported to interact with SNAPc directly^29,42^. To decipher the mechanism of PIC assembly at human U6 promoter, we built an atomic model by combining human TFIIIB^Brf2^-TATA structure (PDB 5N9G) with ours (Fig. 5c). After molecular dynamic simulation, we observed a potential interface between mini-SNAPc and TFIIIB^Brf2^, where the CC-Rh domain of SNAP190 comes in contact with the C-cyclin domain of Brf2 (Fig. 5c). Particularly, this interface is largely contributed by the “Arch” motif, a semicircular α-helix that only exists in Brf2 to specially interact with SNAPc^39^. To verify whether SNAPc depends on CC-Rh domain to facilitate communication with Brf2, CC-Rh deletion on SNAPc was tested to reduce Brf2-binding capacity by ~70% using pull-down assay (Fig. 5e, f, Supplementary Fig. 13b). This result further supports our model. Besides sharing similar TBP-binding domain, Brf2 and TFIIB have been shown to compete for binding to SNAPc in mutually exclusive fashion^29^. Thus, SNAPc favors to interact with TATA-bound Brf2-TBP complex, with exclusion of TFIIB-TBP dimeric complex^29^. As for Bdp1, it efficiently binds to pre-bent Brf2–TBP–DNA complexes^43^, in which the conserved SANT domain is not observed to come in contact with SNAPc spatially. After combining our model with a yeast Pol III-TFIIIB^Brf1^ structure (PDB 6EU0), we noticed that the NTD of yBdp1 is very close to hSNAPc (Fig. 5d). This model is consistent with biochemical results of the NTD of hBdp1 interacting with SNAPc^43^. However, due to the difference between Bdp1-interface of yBrf1 and hBrf2, how hBdp1 and SNAPc communicate remains to be investigated by further structural studies.

Compared with U6 promoter, the TATA-less snRNA promoters mediate transcription initiation in a more complicated manner. The key point is how TBP is positioned when a TATA-box is absent. A recent study proposed a working model of TFIID-supported promoter recognition on TATA-less Pol II-dependent promoters^44^. The role of TFIID complex is particularly important for TBP positioning because IID-C region can specifically bind to DPE (TFIID-binding element) within related mRNA genes. In TATA-less promoters, TBP functions as a component of TFIID, rather than directly binding to DNA. At the beginning of PIC assembly, the TBP-containing TFIID together with TFIIA assemble as CP-TFIID^ITL^ complex after binding to specific promoter region, such as DPE^44^. However, Pol II-dependent snRNA promoters only contain PSE without DPE. Thus, SNAPc could be essential for Pol II-PIC assembly at the correct position. Here we pointed out a potential model of transcription initiation on TATA-less Pol II snRNA promoters (U1 promoter as an example) (Supplementary Fig. 15). Firstly, SNAPc recognizes U1 PSE of Pol II-dependent snRNA, probably in a conformation different from U6 PSE-bound SNAPc, revealed by ChIP-seq results^29^. Due to DPE lacking in snRNA promoters, the recruitment of Pol II-specific TFs relies on SNAPc. After SNAPc binding to PSE, a direct SNAPc–TBP interaction might contribute to TBP-containing TFIID correct positioning. Another interaction of SNAPc-TFIIA makes TFIIA as a key factor in determining specific Pol II PIC assembly, in which SNAPc-TFIIA eliminates the recruitment of Pol III TFs, such as Brf2 and Bdp1^29^. Hence, CP-TFIID^ITL^ complex can assemble downstream of SNAPc-bound PSE in TATA-less snRNA promoters (Supplementary Fig. 15a). In the process, whether TFIIB is involved and what conformation SNAPc adopts remain elusive, which need to be investigated by more experiments. After CP-TFIID^ITL^ complex formation, the following steps including DNA-bending, module repositioning, and Pol II recruitment might comply with the rules of canonical TATA-less mRNA promoter model^44^. The macromolecular apparatus consists of mini-SNAPc, TFIIA, TBP-TFIID, TFIIB, TFIIF and Pol II (Supplementary Fig. 15b). In the integrated model, the CC-Rh domain of SNAP190 is observed to contact TFIIA. But this interaction still needs to be testified by more investigations. In conclusion, our model may be applied to understand the mechanism of how Pol II PIC assembly on TATA-less promoter without DPE: a specific TF, such as SNAPc, helps TBP-TFIIA-TFIID positioning in the vicinity of TSS.

The boundary between Pol II and Pol III transcription specificity on SNAPc-dependent promoters is not absolute. For instance, three canonical type 3 Pol III-dependent snRNA genes can switch to Pol II transcription with different tendency (RPPH1»U6 > 7SK)^45^. Especially, RPPH1 is unique to efficiently direct both active Pol II or Pol III PIC assembly. This could be relevant to the particular RPPH1 promoter architecture of DSE directly adjacent to PSE^46^. Hence, more investigations are needed to understand PIC assembly and Pols competing usage of these SNAPc-dependent promoters. Our structure of human mini-SNAPc complexed with U6-1 PSE provides a powerful tool to further illustrate the transcription regulation of these genes.

## Methods

### Expression and purification of sub-complexes of SNAPc

Human SNAP19, SNAP43 (full-length or residues 1-268), SNAP50, and SNAP190 (residues 1–505) were co-expressed in insect cells using biGBac method^47^. In brief, SNAP19, N-terminal 6xHis tagged SNAP43, SNAP50 and N-terminal FLAG tagged SNAP190 were cloned into a pLIB vector, separately. The four genes were subsequently sub-cloned into a pBIG1a vector by a Gibson assembly reaction, in which these gene PCR products were connected in series with the linearized pBIG1a vector digested by SwaI. The recombinant baculovirus was generated using the Bac-to-Bac system. One liter of Sf9 cells (1.8 × 10^6^ cells/ml) cultured in SIM SF expression medium (SinoBiological) was infected with 12 ml recombinant virus, and cells were harvested after 60 h at 27 °C with constant shaking. Cell pellet was resuspended and lysed using high pressure homogenizer (JNBIO) in 50 ml lysis buffer of 100 mM KCl, 25 mM HEPES K^+^ (pH 7.6), 12.5 mM MgCl_2_, 10 μM ZnCl_2_, 0.1 mM EDTA (pH 8.0), 3 mM dithiothreitol (DTT), and 0.5 mM phenylmethylsulfonyl fluoride (PMSF). Lysis supernatant was loaded onto anti-FLAG M2 affinity resin (Sigma), and the protein was eluted with 20 ml lysis buffer supplemented with 500 μM FLAG peptides (GeneralBiol). Target protein was further purified and pooled to 5 mg/ml using a Superose 6 increase column (GE Healthcare). The purification process was done at 4 °C. Site-directed mutagenesis were operated within related single-subunit pLIB vectors, which were then sub-cloned into the pBIG1a co-expression vector. These mutants were expressed and purified in the same manner mentioned above.

Strep tagged full-length SNAP19 and MBP tagged SNAP190 (residues 1-143) were co-cloned into a pRSFDuet vector, and SNAP43 (residues 148-268) was cloned into a pMal-c2x Vector with N-terminal MBP tag. The two vectors were co-transformed into Rosetta (DE3) pLysS strain of *Escherichia coli*, which was induced to express the rod complex (mSNAPc^#11^) using 0.3 mM IPTG at 16 °C overnight. Cell pellets were harvested and lysed in the buffer containing 150 mM NaCl, 20 mM HEPES Na^+^ (pH 7.5), and 0.5 mM PMSF by sonication. The targeted protein was purified after Strep-Tactin resin (IBA) and a Superose 6 increase column (GE Healthcare). The whole process was done at 4 °C. Brf2^ΔN^ was expressed and purified as described previously^39^.

### Mass spectrometry analysis

The identification of degraded SNAP43 fragment (the band labeled as asterisk in Supplementary Fig. 1a) was carried out by liquid chromatography-mass spectrometry. Briefly, peptides prepared from in-solution digestion were analyzed by nano system (Thermo Scientific, EASY-nLC1200) coupled with a 1,000,000 FWHM high-resolution Nano Orbitrap Fusion Lumos Tribrid Mass Spectrometer system (Thermo Scientific). The raw files from Orbitrap Lumos were imported into Proteome Discoverer software 2.3 (Thermo Scientific) with the Sequest HT search engine against proteome sequence for data processing. Finally, the target band was identified as the carboxyl terminal degraded SNAP43, with 47.55% coverage (source data are provided as a Source Data file).

### Electrophoretic mobility shift assay

HPLC-grade oligonucleotides equivalent of human U6-1 PSE positive strands were synthesized with 5’-FAM label, and their complementary strands had no modified nucleotides (GeneralBiol). The sequences of wild type and mutated DNAs were shown in the relevant figures. The DNA duplexes were formed by slow cooling after heating at 95 °C for 3 minutes. The 25 bp dsDNA was mixed with increasing amounts of mini-SNAPc in a 10 μl reaction at room temperature (RT) for 30 min. The interaction buffer contains 100 mM KCl, 25 mM HEPES K^+^ (pH 7.6), 12.5 mM MgCl_2_, 10 μM ZnCl_2_, 0.1 mM EDTA (pH 8.0), 10% glycerol, 3 mM DTT, and 0.5 mM PMSF. The final concentration of DNA was 50 nM. The reaction products were loaded onto 6% polyacrylamide gels and resolved by electrophoresis in 1x Tris Borate EDTA (TBE) running buffer at 4 °C and 40 Volt. Gels were imaged on the Amersham Imager 680 (GE Healthcare) and band visualization was carried out using ImageQuant TL version 8.2. For quantitative analysis, the mean and standard deviation were calculated based on three independent experiments. The data was plotted using GraphPad Prism 8.

### Cryo-EM sample preparation and data collection

6.7 μM mSNAPc^#2^ was incubated with 35 bp human U6-1 PSE dsDNA at a 1: 2.5 molar ratio in buffer containing 100 mM KCl, 25 mM HEPES K + (pH 7.6), 12.5 mM MgCl_2_, 10 μM ZnCl_2_, 0.1 mM EDTA (pH 8.0), 3 mM DTT, and 0.5 mM PMSF for 30 min at 4 °C. Subsequently, 3.5 μL of sample was applied onto a glow-discharged holey carbon grid (Quantifoil R1.2/1.3 300 M Au). The grid was immediately blotted for 3 s with a blot force of 2 at 4 °C with 100 % humidity and plunged into liquid nitrogen-cooled ethane using a Vitrobot Mark IV (Thermo Fisher). Micrographs were acquired on a 300 kV Titan Krios microscope (Thermo Fisher) with a K2 Summit direct electron detector (Gatan) using SerialEM^48^. Images were recorded at ×130,000 magnification and calibrated super-resolution pixel size 0.538 Å/pixel. Each 6.95 s movie was dose-fractionated into 40 frames and contained a total dose of 60 electrons per Å^2^. For the sample, a total of 6,221 images were collected with a defocus range from 1.0 μm to 2.5 μm.

### Cryo-EM image processing and structure determination

All dose-fractioned movies were motion-corrected and dose-weighted using MotionCor2^49^. CTF estimation, 2D classification, 3D classification and refinements were all performed in cryoSPARC^50^. For the dataset of DNA-bound complex, approximately 4937 micrographs were selected based on the fitted resolution better than 4 Å as estimated by CTFFIND4^51^. A total of 650,462 particles were auto-picked using blob picker and extracted with a binning factor of 2, resulting in a box size of 150 pixels. Templates were selected from two rounds of 2D classification result of blob picked particles, and a total of 1,923,520 particles were picked using template-based automatic particle picking and extracted with a box size of 150 pixels after binning by 2. A total of 231,274 particles were selected after 2D classification based on complex integrity. This particle set was used for Ab-Initio reconstruction with three classes, which were then used as 3D volume templates for heterogeneous refinement. A subset of 67,058 particles from the class showing clear structural features was selected and re-extracted without binning and with a box size of 300 pixels, which was then subjected to Homogeneous Refinement, Local Refinement and Non-uniform Refinement, giving rise to a 3.49 Å density map. The local resolution map was carried out by Relion^52^.

The de novo model was manually built using COOT^53^ based on the predicted structures by trRosetta server^54^. At the current resolution, the density map of DNA phosphate groups is clear to be positioned easily. Hence, we firstly determined the phosphate backbone of 24 bp ideal B-form dsDNA. Since the sizes of base density are different, we can distinguish between pyrimidine (T or C) and purine (A or G) clearly. We generated a series of PSE models with different positions (the current PSE model and derived models by shifting upstream or downstream of 1, 2 or 3 bases, respectively) or orientations (reversed from 5’ to 3’ end). By evaluating the density map sizes of bases, we confirmed that the PSE at the current location is the only correct model (the evaluation of PSE assignment in Supplementary Fig. 16). After PSE location was determined, several predicted compact domains such as Rb~Rd MYB repeats of SNAP190, the β-barrel domain and Wedge domain of SNAP50, and the NTD of SNAP43 were docked into the density map with slight discrepancy. The initial model was refined using the “real-space refinement” package of PHENIX version 1.18.2^55^. Other parts of model were stepwise built into the map with multiple rounds of manual model-building by COOT and automatic refinement by PHENIX. The final structure validation was performed using “Comprehensive validation (cryo-EM)” module of PHENIX. The statistics of the 3D reconstruction and structure refinement are summarized in Table S2. The cryo-EM density maps were calculated with UCSF Chimera^56^, and structure-related figures were prepared with PyMol (http://www.pymol.org) or UCSF ChimeraX^57^.

### Co-purification of truncated Mini-SNAPc using anti-flag affinity chromatography

Four SNAP190 constructs including SNAP190^(140-505)^ (extreme NTD deletion), SNAP190^(180-505)^ (Region I deletion), SNAP190^(140-296+4GS+345-505)^ (Region III substitution) and SNAP190^(180-296+4GS+345-505)^ (Region I deletion and Region III substitution) with N-terminal FLAG tag, three SNAP50 constructs of SNAP50^(1-411)^ (full length), SNAP50^(1-351)^ (wedge domain deletion), and SNAP50^(141-411)^ (lasso domain deletion) with N-terminal Strep tag, and N-terminal His-TEV tagged NTD domain of SNAP43 were cloned into the pLIB vector separately. These clones were then combined into the co-expression pBIG1a vector accordingly to generate different partial Mini-SNAPc complexes designated as mSNAPc^#3^ ~mSNAPc^#9^ shown in Supplementary Table 1. These complexes were expressed using the same expression strategy applied to the cryo-EM samples. Finally, 100–200 ml Sf9 cells infected by recombinant baculovirus were harvested and lysed for each complex. The supernatants after centrifugation were loaded onto anti-FLAG M2 resins (Sigma) and targeted proteins were purified after flow-through of 2 ml lysis buffer containing 500 μM FLAG peptides. The samples were prepared immediately for SDS-PAGE and Western blotting. The nitrocellulose membranes (GE Healthcare) were blocked in TBS-T buffer containing 5% milk for at least 1 h at RT. Primary antibodies against FLAG (F3156, Sigma, 1:400), Strep (2-1509-002, IBA, 1:10000), and TEV cleavage site (PA1-119, Thermo Fisher, 1:1000) were incubated at 4 °C overnight. After wash by TBS-T three times, the membranes were incubated with different secondary antibodies, HRP-anti mouse IgG or HRP-anti rabbit IgG (abs20001 or abs20002, Absin, 1:10000) at RT for 1 h. Detection was achieved with AI680RGB Amersham Imager 680.

### Surface plasmon resonance analysis

The protein-DNA kinetics were investigated using Biacore T200 (GE Healthcare). The running buffer composed of 100 mM KCl, 12.5 mM MgCl_2_, 10 μM ZnCl_2_, 0.1 mM EDTA (pH 8.0), and 0.05% surfactant P20 was prepared, vacuum filtered, and degassed immediately prior to use. WT and mutants of mSNAPc^#2^ were immobilized on a CM5 sensorchip via amine groups in 10 mM sodium acetate buffer (pH 5.5) to a level of around 10000 response units. Serial dilutions of the annealing 25 bp human U6-1 PSE duplex with HPLC grade (GeneralBiol) were flowed through with a concentration ranging from 2000 to 31.25 nM at 25 °C. The resulting data were fit to a 1:1 binding model using Biacore Evaluation Software (GE Healthcare).

### Molecular dynamics simulation of mini-SNAPc/TFIIIB/U6 promoter

To create an initial model of mini-SNAPc/TFIIIB/U6 promoter, we first generated two separate models of mini-SNAPc/PSE and TFIIIB/TATA. Namely, we extended the downstream DNA of our mini-SNAPc/PSE structure to the position -30 using ideal B-form dsDNA model in COOT. In TFIIIB/TATA structure (PDB ID 5N9G), the original U6-2 sequence was replaced by human U6-1 sequence. Subsequently, the duplex DNAs of two models from the position -34 to -31 were superposed in COOT. Taking the -35th base pair as the boundary, the upstream DNA bound by mini-SNAPc and the downstream DNA bound by TFIIIB were merged to be an initial model of mini-SNAPc/TFIIIB/U6 promoter.

MD simulation was performed using the AMBER 14 software package, employing the Amber14SB force field^58^ for the protein, ff99bsc0_chiOL3^59^ for RNA and the TIP3P model for water molecules. The parameters for zinc were obtained from the Zinc AMBER Force Field (ZAFF)^60^. Then, the model of hU6 promoter bound by mini-SNAPc and TFIIIB was neutralized with Na^+^ counterions and solvated with explicit water in a rectangular periodic box with 10.0 Å buffer using AmberTools 15. All other parameters were default values. After a series of minimizations and equilibrations^61^, MD simulations were performed on GPUs using the CUDA version of PMEMD^62,63^.

### Pull-down assay

First, purified mSNAPc^#2^ and mSNAPc^#10^ (2 μM) were incubated with 35 bp U6-1 dsDNA in the same condition as EMSA. Then the protein-DNA complexes were immobilized on anti-FLAG M2 resins (Sigma), and incubated with same amount of Brf2^ΔN^ for 1 h at 4 °C in the buffer of 20 mM HEPES at pH 7.5, 300 mM NaCl, 0.1% Tween-20. After washed four times, and the samples were released from beads using 50 μl 0.15 M Glycine buffer at pH 3.5. N-terminal FLAG tagged SNAP190^1-505^ and SNAP190^1-505(ΔCC-Rh)^ were detected by FLAG antibody (F3156, Sigma, 1:400) and Brf2^ΔN^ was detected by Brf2 antibody (12056-1-AP, Proteintech, 1:1000). For quantitative analysis, the mean and standard deviation were calculated on the basis of three independent experiments. The data were plotted with GraphPad Prism 8.

### Reporting summary

Further information on research design is available in the Nature Portfolio Reporting Summary linked to this article.

## Supplementary information

Supplementary Information

Reporting Summary

## Data Availability

The data that support this work are available from the corresponding authors upon reasonable request. The cryo-EM density map and the atomic model have been deposited to the Electron Microscopy Data Bank (EMDB) and Protein Data Bank (PDB) under the accession codes EMD-33477 and 7XUR, respectively. Source data are provided with this paper.

## Source data

source data
