# Supplementary material for: Structural basis of human SNAPc recognizing proximal sequence element of snRNA promoter
Source: Nat Commun. 2022 Nov 11;13:6871. doi: 10.1038/s41467-022-34639-1 (PMC9652321; doi:10.1038/s41467-022-34639-1)
Supplement: Supplementary file 1 — Supplementary Information [file 41467_2022_34639_MOESM1_ESM.pdf]

Supplementary Information

Structural basis of human SNAPc recognizing proximal sequence element of snRNA promoter

Jianfeng Sun[1,2,3,8,*], Xue Li[1,8], Xuben Hou[4], Sujian Cao[1], Wenjin Cao[1], Ye Zhang[1], Jinyang Song[1], Manfu Wang[5], Hao Wang[1], Xiaodong Yan[5], Zengpeng Li[6], Robert G. Roeder[3,*], Wei Wang[1,7,*]

[1]Advanced Medical Research Institute, Cheeloo College of Medicine, Shandong University, Jinan 250012, China.

[2]Department of Biochemistry and Molecular Biology, School of Basic Medical Sciences, Cheeloo College of Medicine, Shandong University, Jinan 250012, China.

[3]Laboratory of Biochemistry and Molecular Biology, The Rockefeller University, New York 10065, USA.

[4]School of Pharmaceutical Sciences, Cheeloo College of Medicine, Shandong University, Jinan 250012, China.

[5]Wuxi Biortus Biosciences Co. Ltd., Jiangyin 214437, China.

[6]Third Institute of Oceanography, Ministry of Natural Resources, Xiamen 361005, China.

[7]Interventional Medicine Department, The Second Hospital, Cheeloo College of Medicine, Shandong University, Jinan 250033, China.

[8]These authors contributed equally: Jianfeng Sun and Xue Li.

*To whom correspondence may be addressed. Email: sunjf@sdu.edu.cn; roeder@rockefeller.edu; weiwang007@sdu.edu.cn

This PDF file includes:

Supplementary Figs. 1-16

Supplementary Tables 1-2

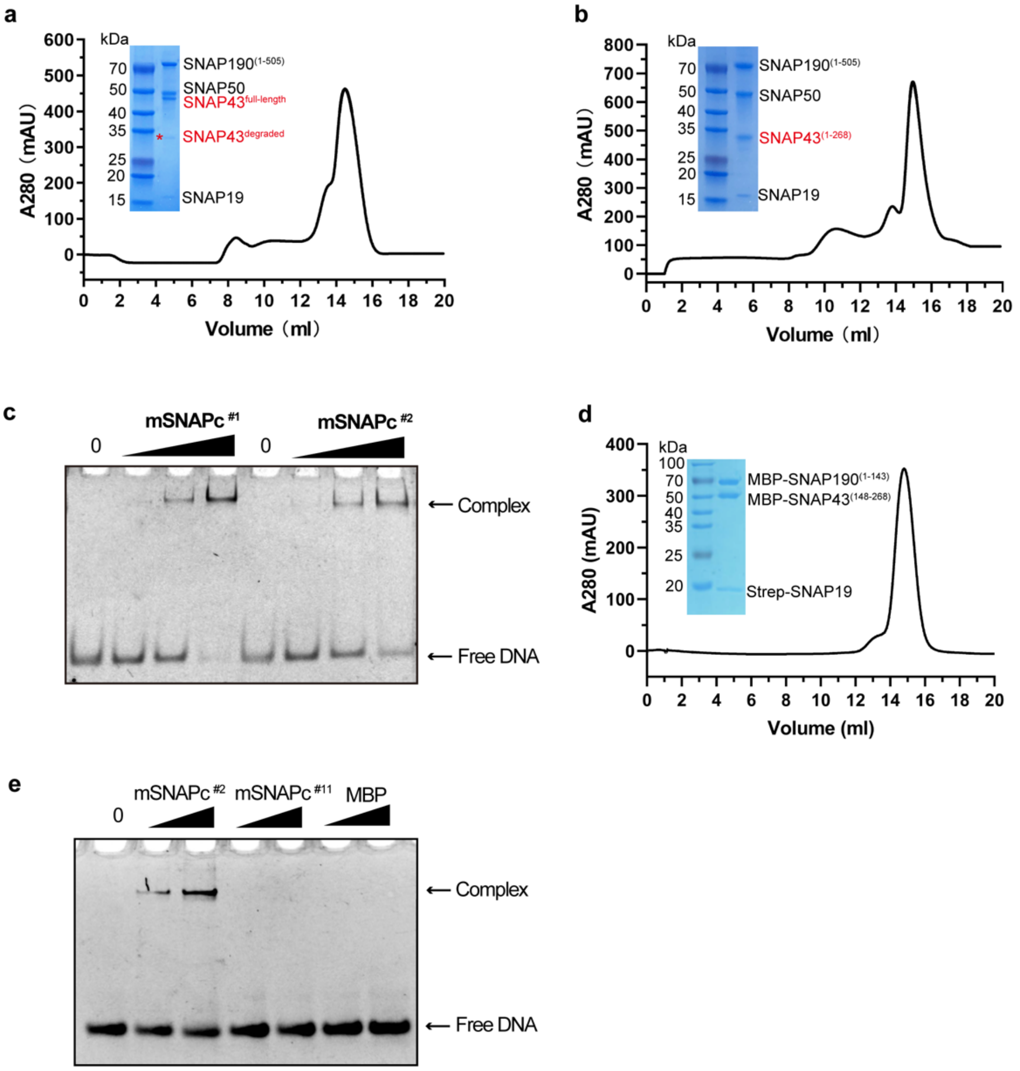

Supplementary Fig. 1 | Mini-SNAPc reconstitution and DNA-binding capacity analysis. a Size exclusion chromatography of mSNAPc$^{\#1}$ with SDS-PAGE analysis. **b** Size exclusion chromatography of mSNAPc$^{\#2}$ with SDS-PAGE analysis. **c** Comparison of PSE-binding activity between mSNAPc$^{\#1}$ and mSNAPc$^{\#2}$ by EMSA. 0, 200, 600 and 1200 nM proteins were titrated into 50 nM 25 bp hU6-1 dsDNA, separately. **d** Size exclusion chromatography of mSNAPc$^{\#11}$ (the rod module) with SDS-PAGE analysis. **e** EMSA showed that mSNAPc$^{\#11}$ has no PSE-binding ability compared with mSNAPc$^{\#2}$. 200 and 800 nM proteins were applied here. Purified MBP protein was used as a control, with the same concentration gradients. All experiments above were repeated independently three times with similar results.

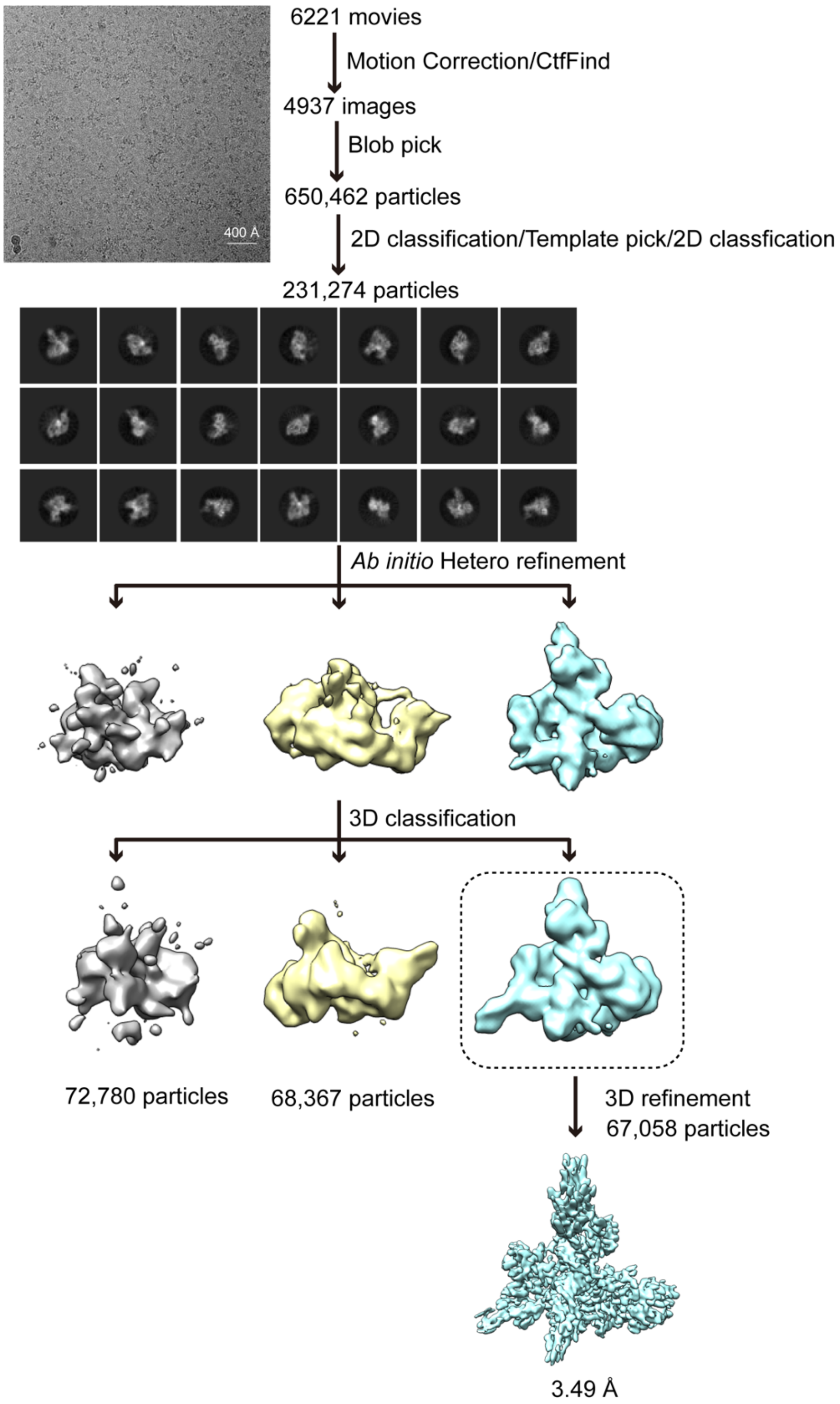

Supplementary Fig. 2 | Single-particle cryo-EM image processing workflow.

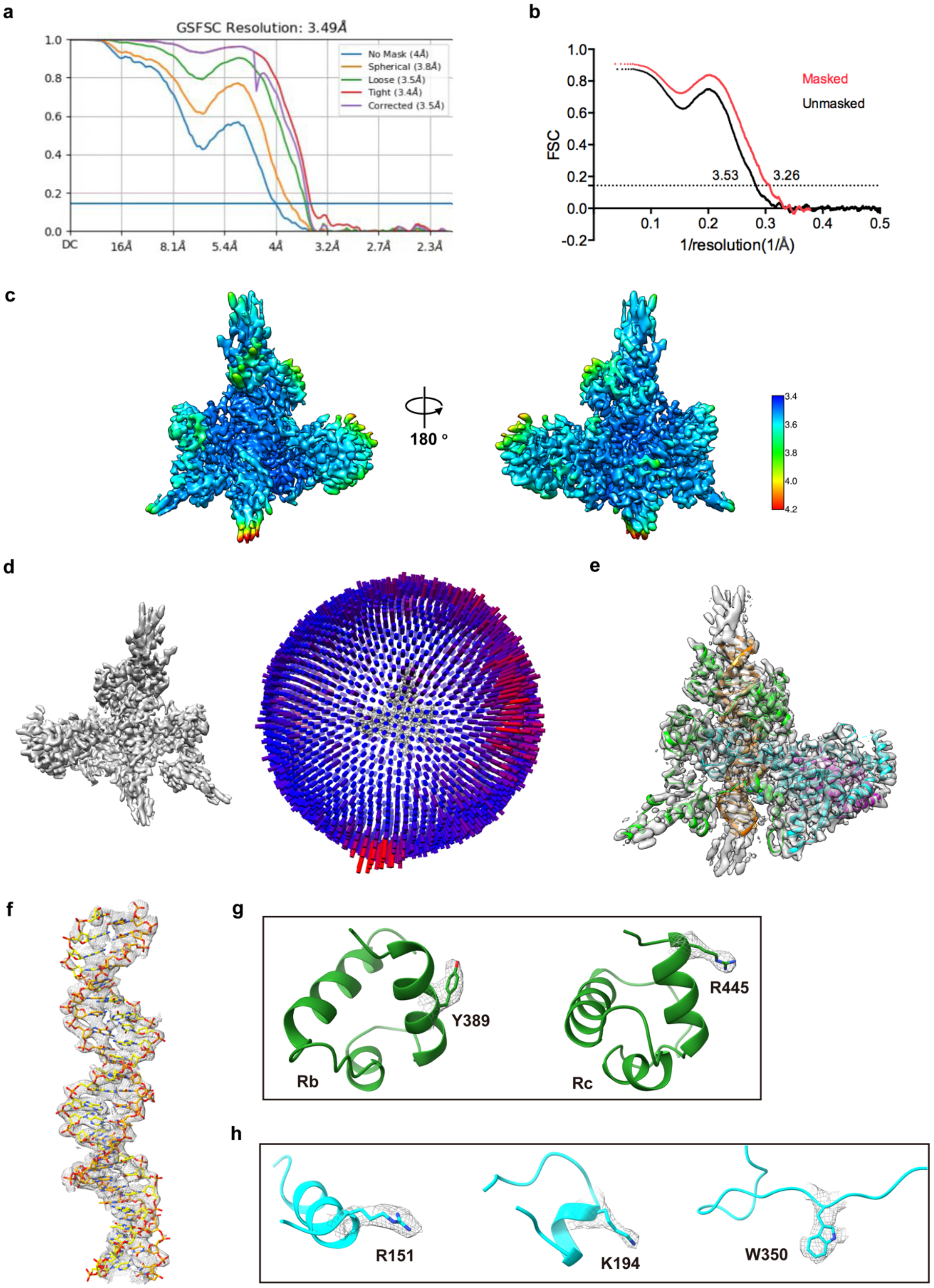

a
GSFSC Resolution: 3.49Å
No Mask (4Å)
Spherical (3.8Å)
Loose (3.5Å)
Tight (3.4Å)
Corrected (3.5Å)
b
FSC
Masked
Unmasked
3.53
3.26
1/resolution(1/Å)
c
180 °
d
e
f
g
Y389
Rb
R445
Rc
h
R151
K194
W350

Supplementary Fig. 3 | The analysis of single-particle cryo-EM data and the final model. **a** Fourier shell correlation (FSC) curve of the final 3D reconstruction. **b** FSC curves between the map and model. **c** Local-resolution cryo-EM density map generated by Relion. **d** Angular distribution of the particles contributing to the final reconstruction of mini-SNAPc/hU6-1 complex. **e** The cryo-EM density map docked by the final structure model. **f** Atomic model of 24 bp DNA duplex displayed inside the segmented cryo-EM density. **g** The key residues of SNAP190, namely Y389 of Rb and R445 of Rc are shown as sticks covered with cryo-EM densities. **h** The key residues of SNAP50, namely R151 of Motif A, K194 of Motif B, W350 of Motif C are shown as sticks covered with cryo-EM densities.

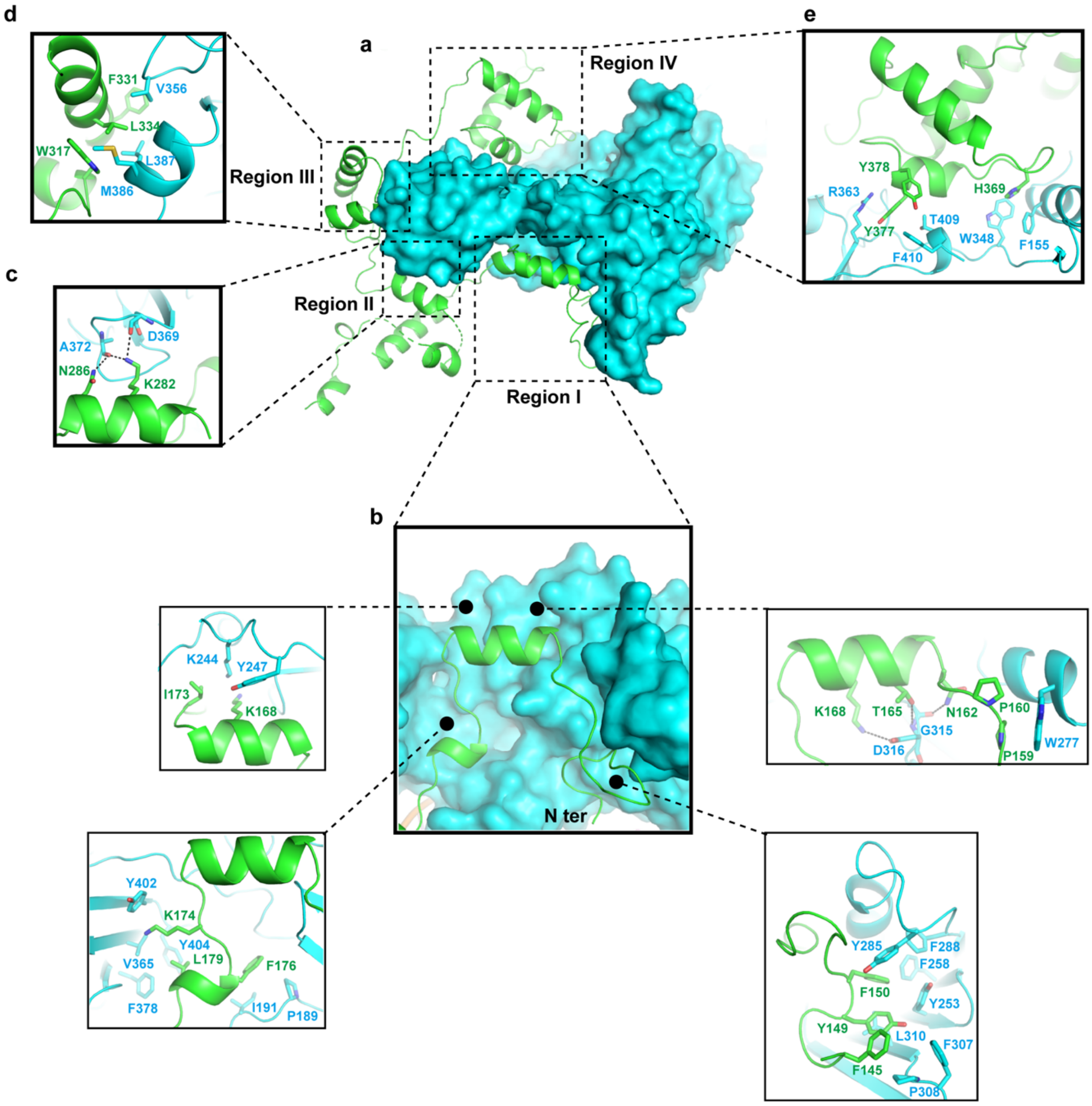

Supplementary Fig. 4 | Structural views of interface between SNAP190 and SNAP50. a Four regions of SNAP190$^{(1\text{-}505)}$ encircle the wedge domain of SNAP50. **b** Region I is mainly docked on the surface of the β-barrel domain of SNAP50. **c** Hydrogen bonds are formed between Region II and SNAP50. **d** Region III (Ra) mainly depends on a hydrophobic surface to interact with SNAP50. **e** The contact between Region IV (Rb) and SNAP50. All residues participating in subunit-subunit interactions are shown as stick models.

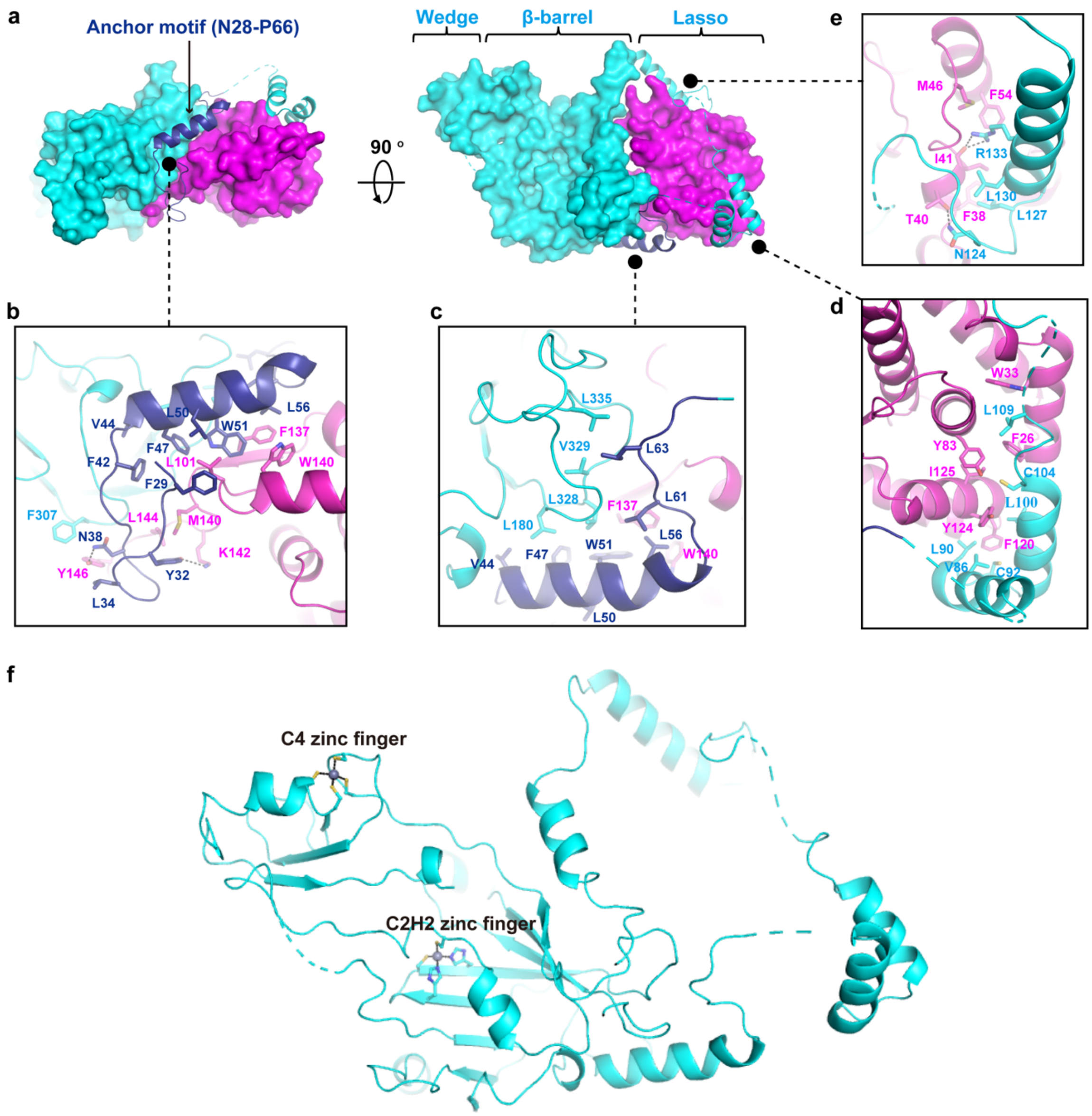

Supplementary Fig. 5 | SNAP50 encircles the NTD of SNAP43 in a lasso-like mode. **a** An anchor motif of SNAP50 mediates the interaction between the β-barrel domain of SNAP50 and the NTD of SNAP43. **b**, **c** Two different structural views of detailed residues involved in anchor motif docking. **d**, **e** Other two fragments of the lasso domain of SNAP50 interact with the NTD of SNAP43. **f** Two zinc fingers function as an architectural component of SNAP50.

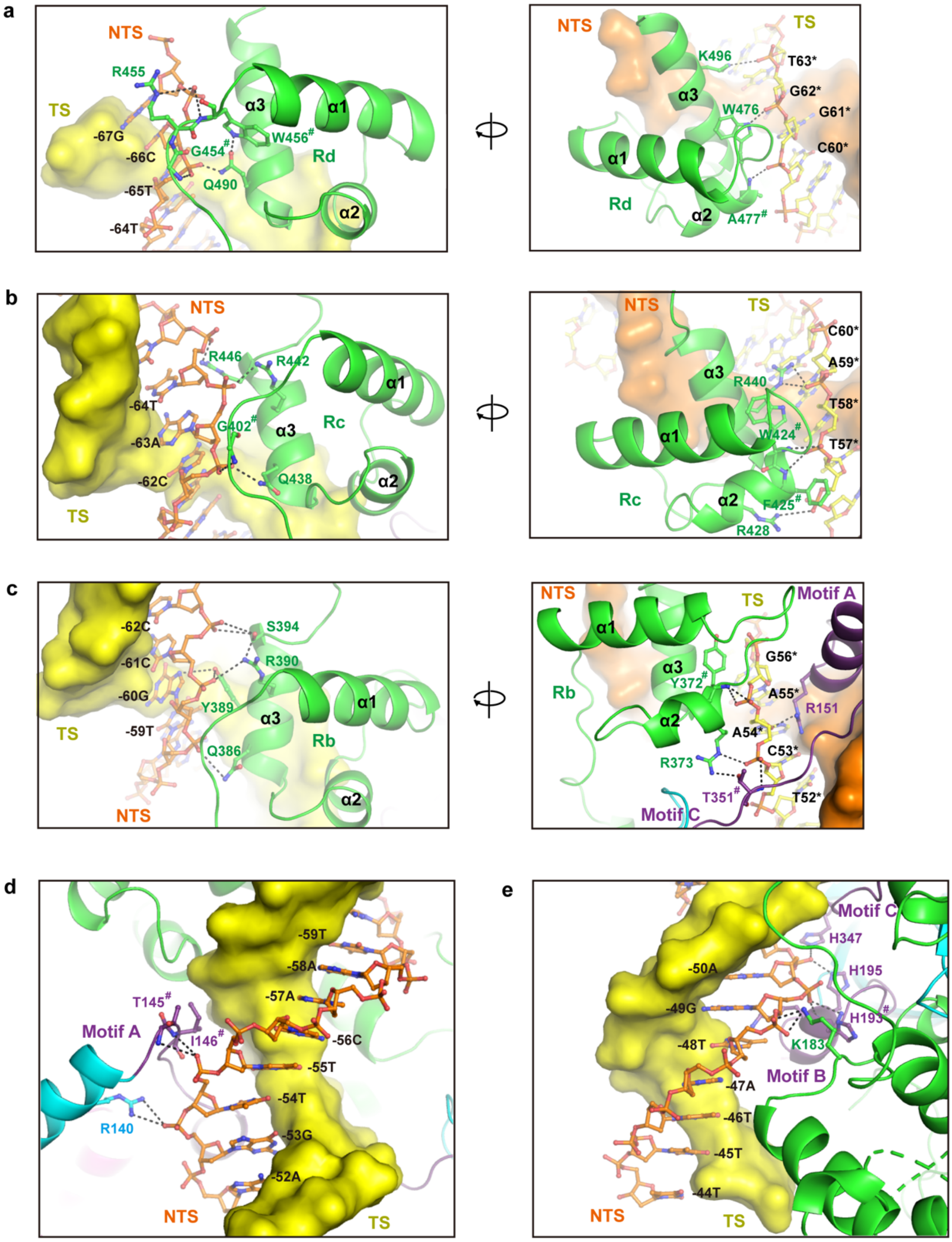

a
NTS
R455
TS
-67G
-66C
G454#
W456#
α3
α1
Rd
Q490
-65T
-64T
α2
NTS
TS
K496
T63*
α3
G62*
W476
G61*
C60*
α1
Rd
α2
A477#
b
NTS
R446
R442
α1
-64T
G402#
α3
Rc
-63A
-62C
Q438
α2
TS
NTS
TS
C60*
A59*
α3
R440
T58*
α1
W424#
T57*
Rc
α2
F425#
R428
c
-62C
S394
-61C
R390
-60G
Y389
α1
α3
TS
-59T
Rb
Q386
α2
NTS
NTS
α1
TS
Motif A
G56*
α3
Rb
Y372#
A55*
α2
R151
A54*
C53*
R373
T351#
T52*
Motif C
d
-59T
-58A
-57A
T145#
Motif A
I146#
-56C
-55T
-54T
R140
-53G
-52A
NTS
TS
e
Motif C
H347
-50A
H195
-49G
H193#
-48T
K183
Motif B
-47A
-46T
-45T
-44T
NTS
TS

Supplementary Fig. 6 | Non-specific interactions between mini-SNAPc and sugar-phosphate backbone of dsDNA. a Rd of SNAP190 uses G454, R455, W456 and Q490 to form hydrogen bonds with phosphate backbone of NTS (left panel), and W476, A477 and K496 to interact with the phosphate groups of TS (right panel). **b** Residues on Rc of SNAP190 form polar interaction with the phosphate groups on -64T and -62C of NTS (left panel) and T57*, T58*, and A59* of TS (right panel). **c** Rb of SNAP190 utilizes Q386, R390, and S394 to form hydrogen bonds with phosphate groups of NTS (left panel), and Y372 and R373 to interact with TS (right panel). R151 and T351 of SNAP50 are also involved. **d**, **e** The non-specific interaction of SNAP50-PSE focuses on a hydrogen-bond network formed by residues of three key motifs of SNAP50 and the phosphate backbone of NTS.

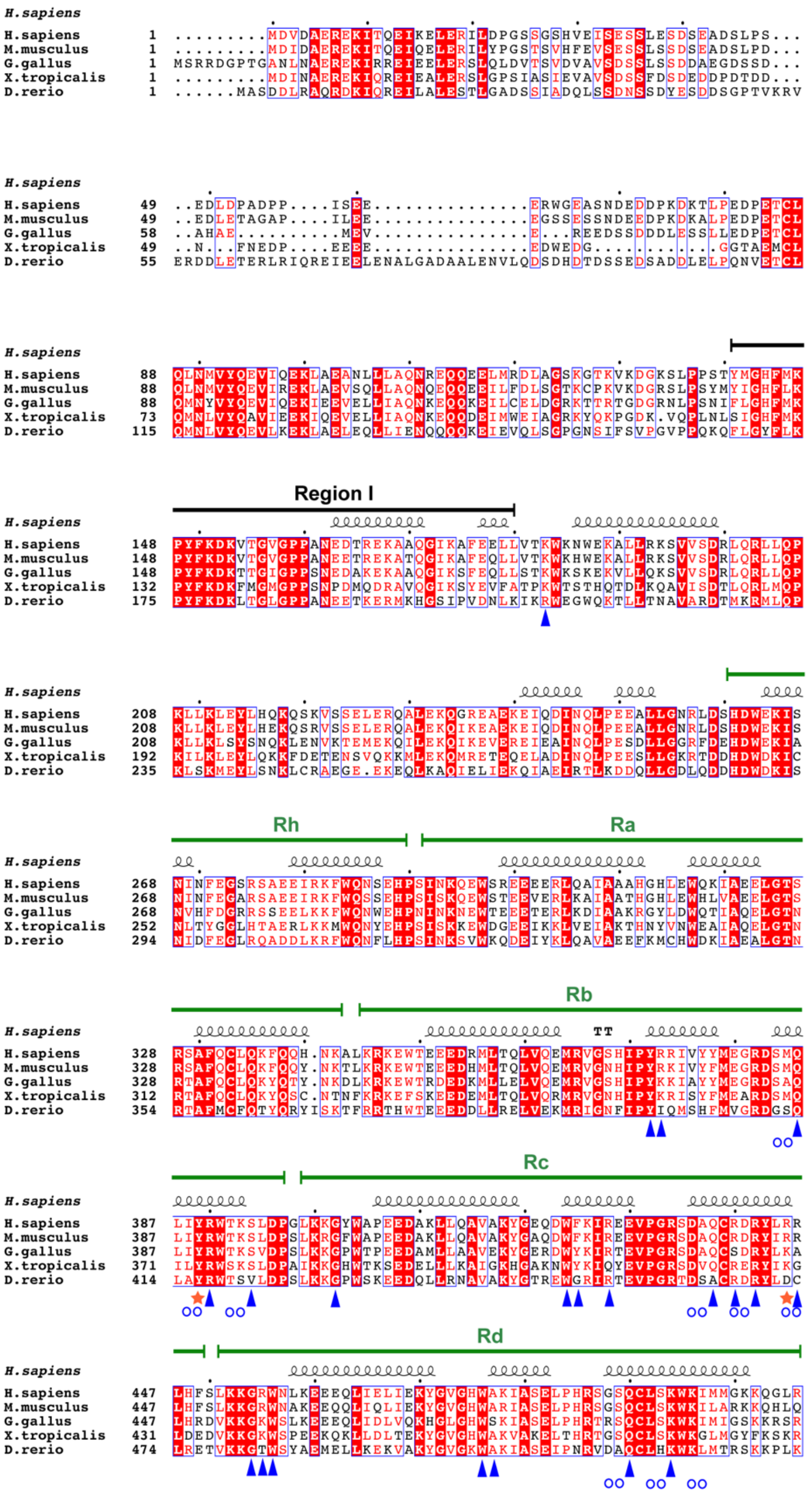
Region I
Rh
Ra
Rb
Rc
Rd

Supplementary Fig. 7 | Sequence alignment of the NTD of five SNAP190 homologs from *H. sapiens*, *M. musculus*, *G. gallus*, *X. tropicalis*, and *D. rerio*. Two key residues guiding nucleotide-specific recognition are highlighted as orange stars. Residues involved in non-specific interaction with sugar-phosphate backbone of DNA are labeled as blue triangles. Residues of MYB repeats pointing toward major groove of PSE are indicated as blue circles.

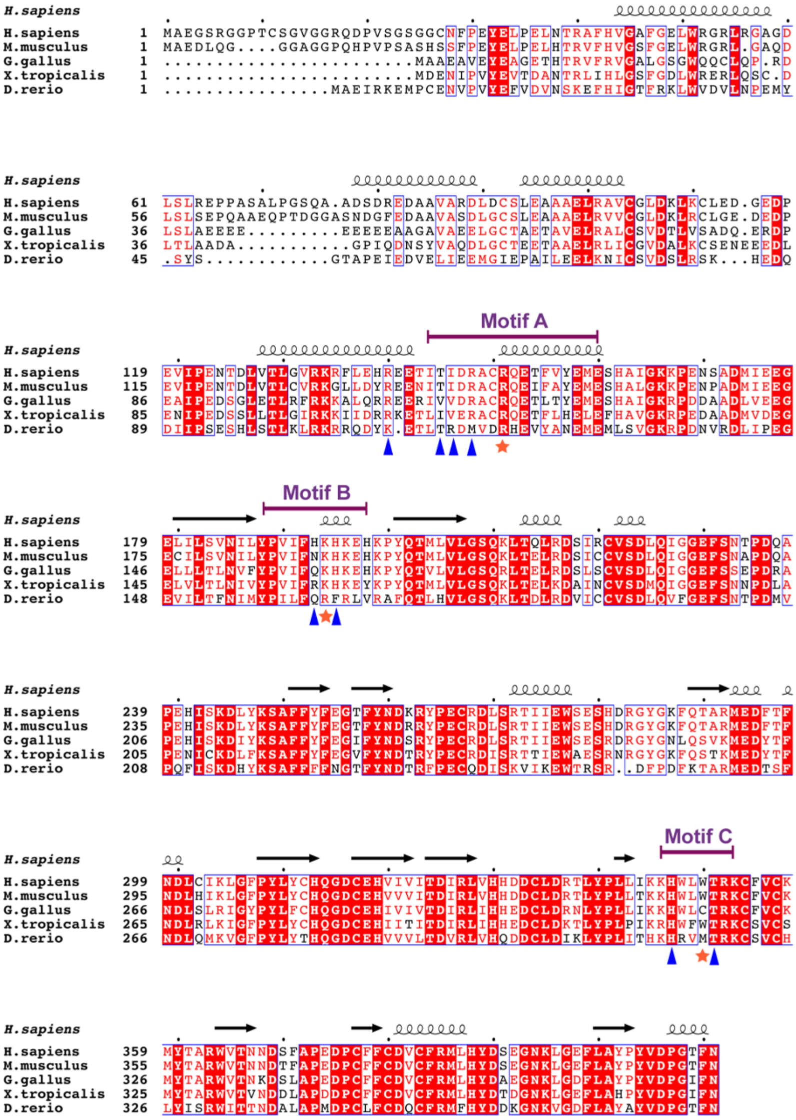

Supplementary Fig. 8 | Sequence alignment of five SNAP50 homologs from *H. sapiens*, *M. musculus*, *G. gallus*, *X. tropicalis*, and *D. rerio*. Three key residues guiding nucleotide-specific recognition are highlighted as orange stars. Residues involved in non-specific interaction with sugar-phosphate backbone of DNA are labeled as blue triangles.

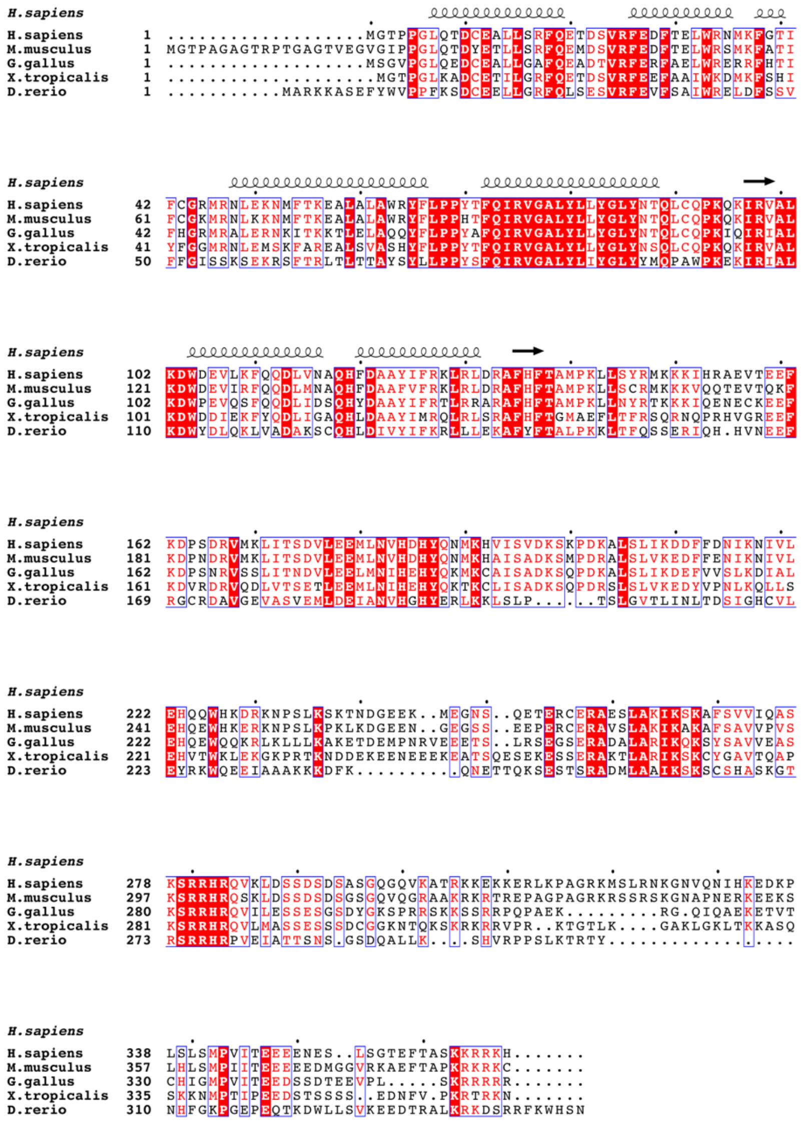

Supplementary Fig. 9 | Sequence alignment of five SNAP43 homologs from *H. sapiens*, *M. musculus*, *G. gallus*, *X. tropicalis*, and *D. rerio*.

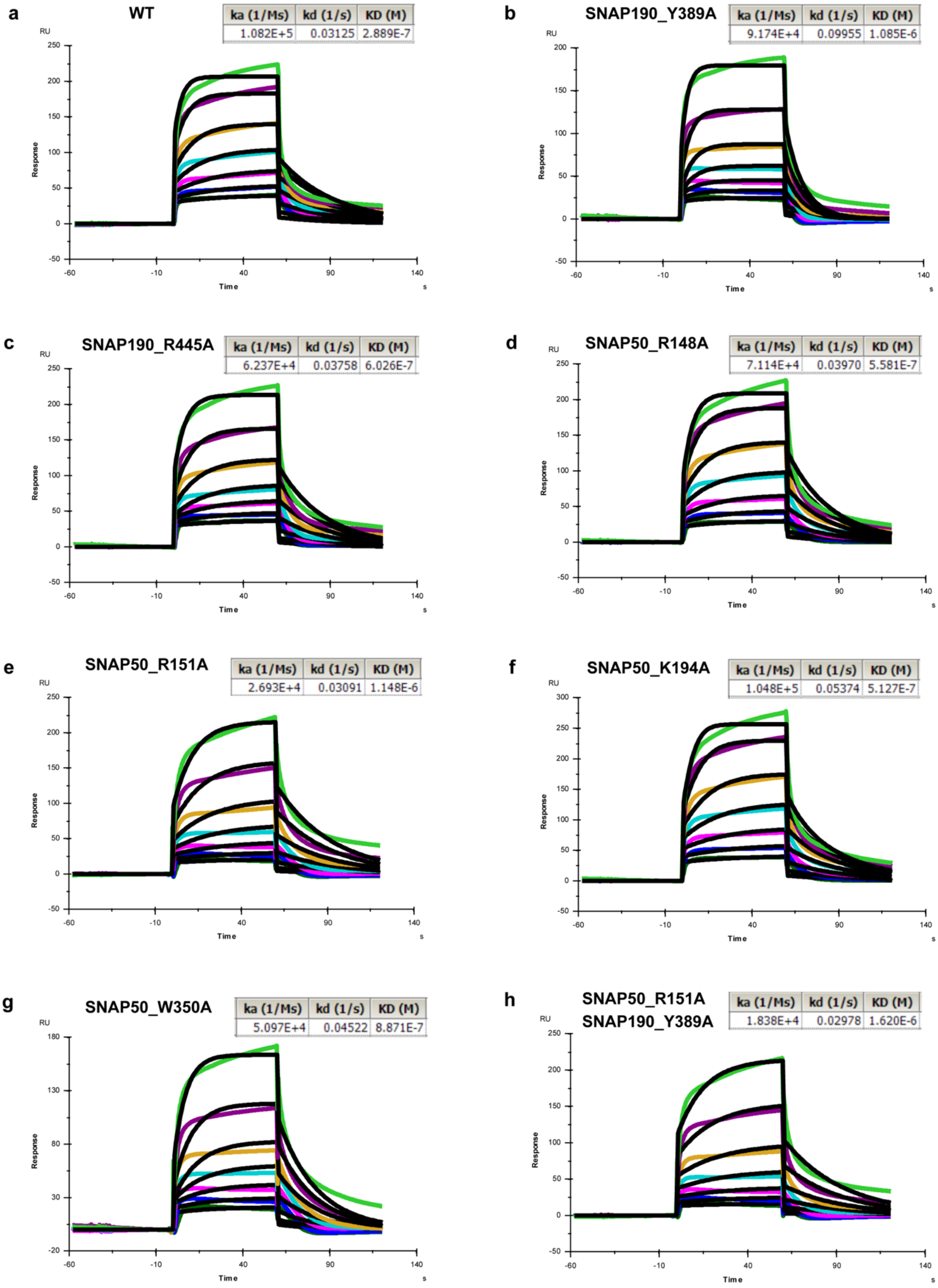
a
WT
ka (1/Ms) kd (1/s) KD (M)
1.082E+5 0.03125 2.889E-7
b
SNAP190_Y389A
ka (1/Ms) kd (1/s) KD (M)
9.174E+4 0.09955 1.085E-6
c
SNAP190_R445A
ka (1/Ms) kd (1/s) KD (M)
6.237E+4 0.03758 6.026E-7
d
SNAP50_R148A
ka (1/Ms) kd (1/s) KD (M)
7.114E+4 0.03970 5.581E-7
e
SNAP50_R151A
ka (1/Ms) kd (1/s) KD (M)
2.693E+4 0.03091 1.148E-6
f
SNAP50_K194A
ka (1/Ms) kd (1/s) KD (M)
1.048E+5 0.05374 5.127E-7
g
SNAP50_W350A
ka (1/Ms) kd (1/s) KD (M)
5.097E+4 0.04522 8.871E-7
h
SNAP50_R151A
SNAP190_Y389A
ka (1/Ms) kd (1/s) KD (M)
1.838E+4 0.02978 1.620E-6
RU
Response
Time
s

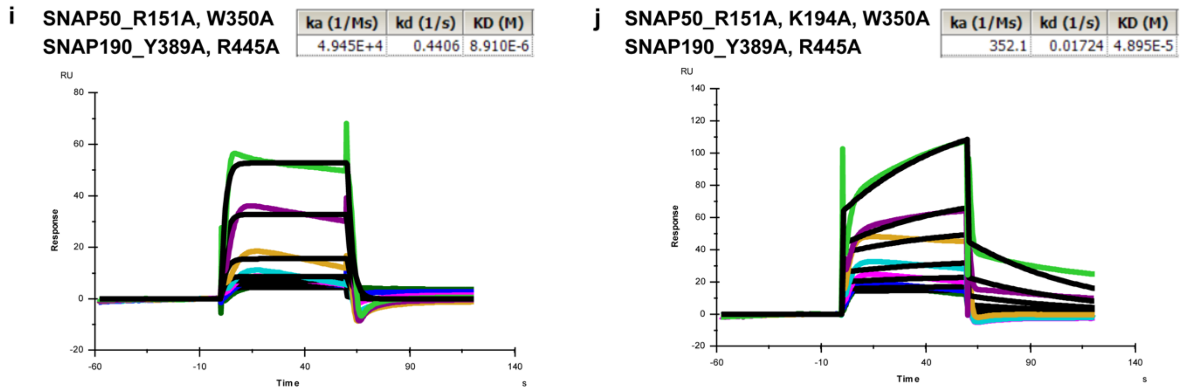

Supplementary Fig. 10 | Surface plasmon resonance sensorgram. Binding kinetics of 25 bp hU6-1 PSE duplex with WT and mutated mSNAPc[#2] were tested by SPR. Colored lines indicate the experimentally derived curves, and black lines represent best-fitted curves using a 1:1 binding model. ka, association rate constant; kd, dissociation rate constant; KD, dissociation constant.

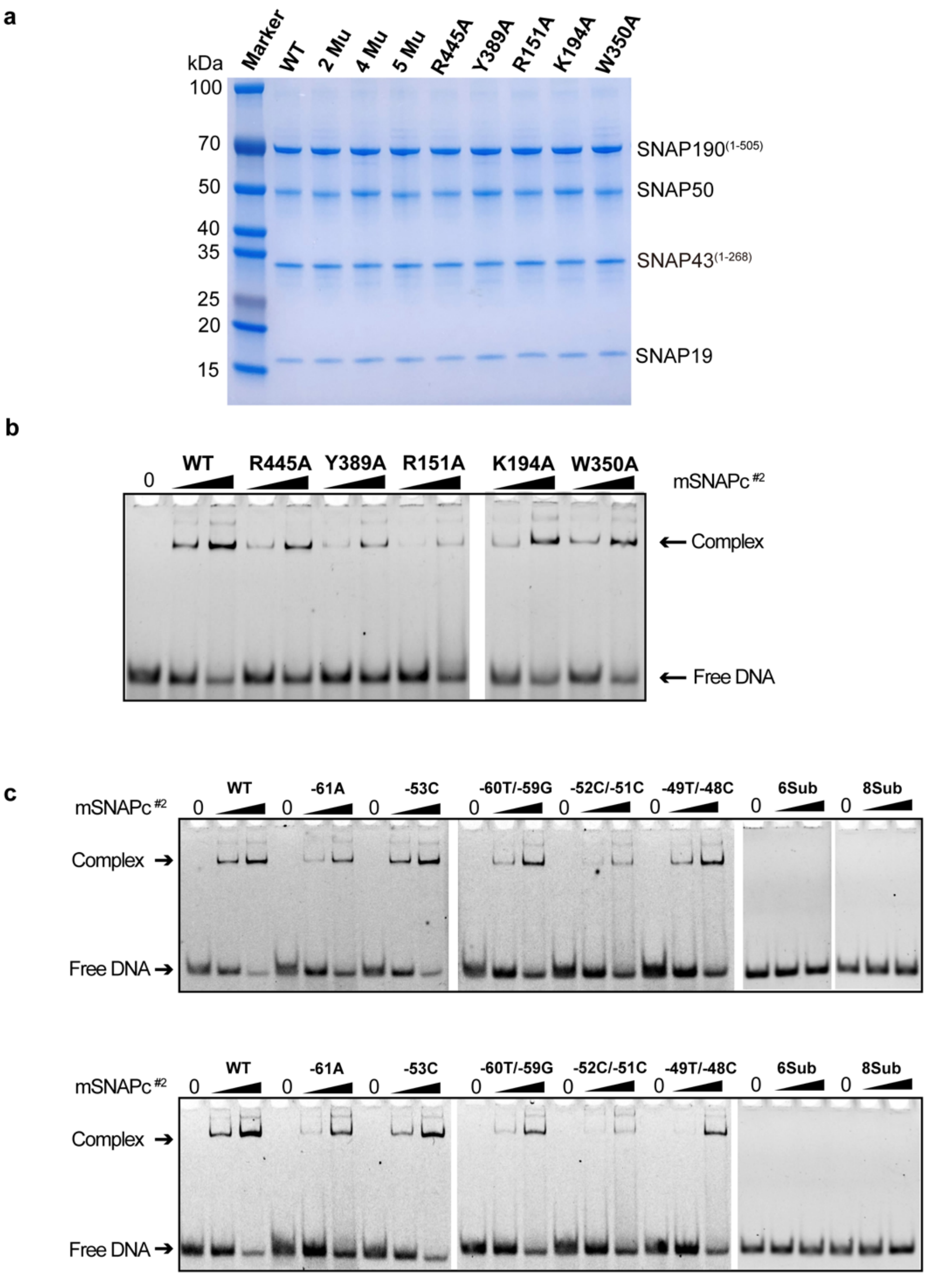

Supplementary Fig. 11 | SNAPc relies on synergistic action of multiple residues for PSE-recognition. a SDS-PAGE analysis of proteins (WT and mutants) used for SPR and EMSA. This experiment was repeated independently three times with similar results. **b** The PSE-binding capacity was tested for WT and five single-point mutated mSNAPc$^{\#2}$. For each experiment, 200 and 1000 nM proteins (WT or mutant) were incubated with 50 nM fluorescently labeled 25 bp DNA probes. This experiment was repeated independently three times with similar results. **c** Twice-repeated EMSAs of mini-SNAPc and seven nucleotide-substituted hU6-1 PSE variants. Together with data from Fig. 4c, they are used for quantitative calculation in Fig. 4d.

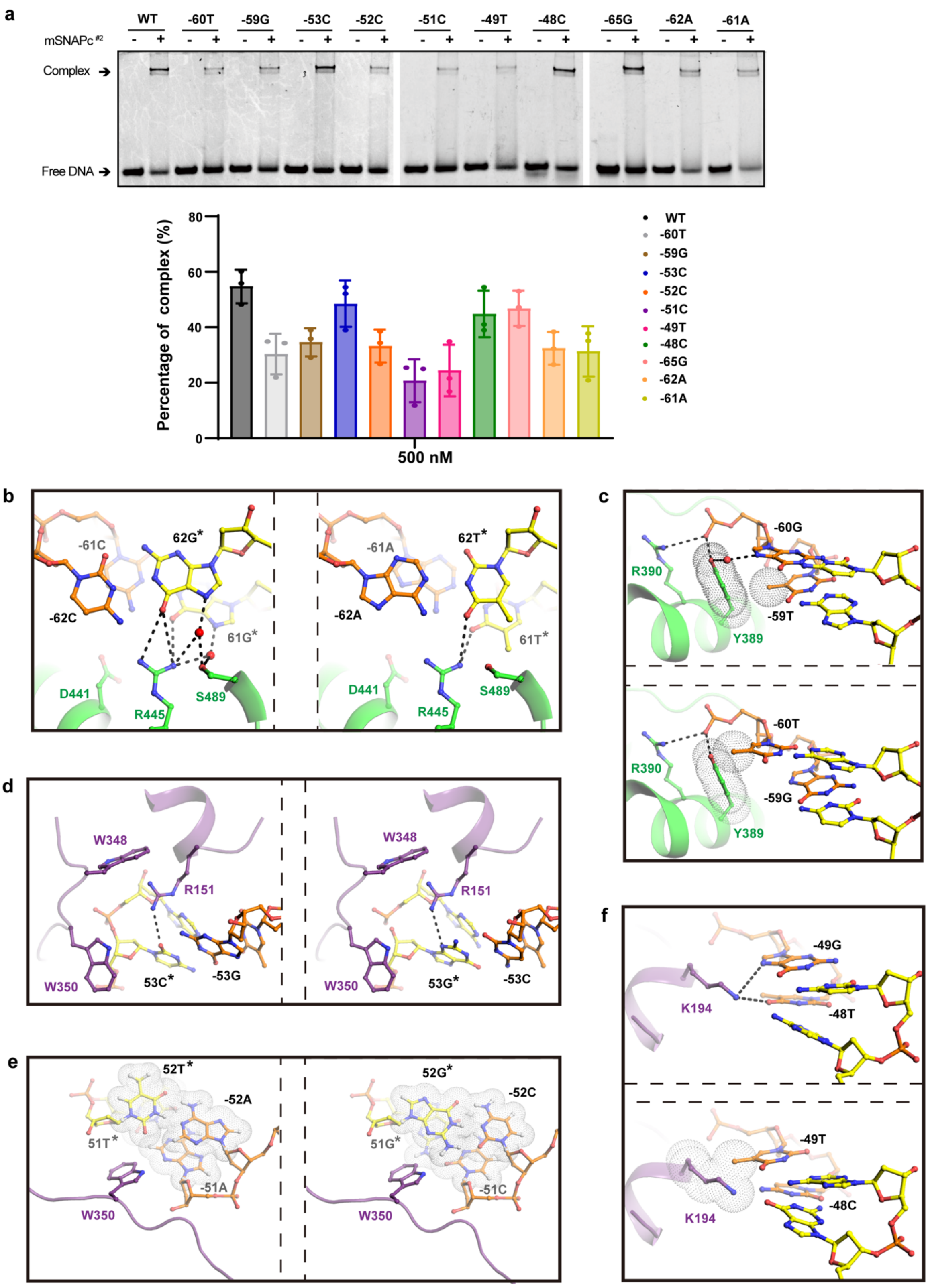

a
WT
-60T
-59G
-53C
-52C
-51C
-49T
-48C
-65G
-62A
-61A
mSNAPc #2
Complex
Free DNA
Percentage of complex (%)
500 nM
b
62G*
-61C
-62C
61G*
D441
R445
S489
62T*
-61A
-62A
61T*
c
-60G
R390
-59T
Y389
-60T
-59G
d
W348
R151
W350
53C*
-53G
53G*
-53C
e
52T*
-52A
51T*
-51A
W350
52G*
-52C
51G*
-51C
f
-49G
K194
-48T
-49T
-48C

Supplementary Fig. 12 | EMSA and structural modeling analyses of single-nucleotide substituted PSEs interacting with mSNAP$^{\#2}$. a EMSA was tested for SNAPc-binding capability of ten single-nucleotide substituted PSEs. The bottom panel is the quantitative analysis based on three independent experiments (other two EMSAs shown in Supplementary Fig. 13a). Data are mean ± SEM (n=3). Source data are provided as a Source Data file. **b** The "AT" pair substitution at positions -62 and -61 might disrupt the potential strong H-bond network among waters, R445, G62*, G61* and S489. **c** Structural analysis showed that the methyl group of substituted -60T could clash with the OH group of Y389, and substituted -59G could attenuate hydrophobic interaction with the aromatic ring of Y389. **d** The hydrogen bond of R151 is maintained, regardless of the substituted GC pair at position -53. W348 and W350 are surrounding R151 to block potential water-mediated hydrogen bonds. **e** When AT pairs are replaced by CG pairs at position -52 and -51, the environment around W350 is more hydrophilic (the N2 atoms of guanosines are close to W350) to hinder W350 from inserting into the minor groove through hydrophobic interaction. **f** The methyl group of substituted -49T could clash with the side chain of K194 so as to break two original hydrogen bonds. Substituted -48C only affect one original hydrogen bonds between K194 and -48T.

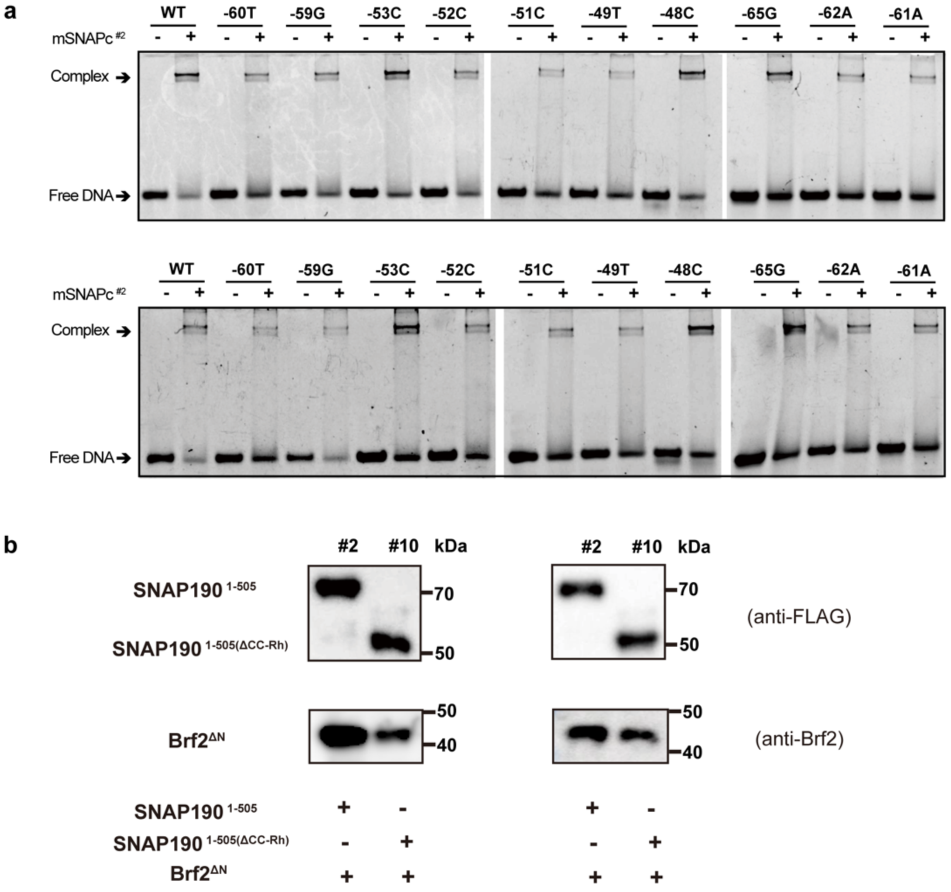

Supplementary Fig. 13 | a Other two repeated EMSAs of single-nucleotide substituted PSEs interacting with mSNAP$^{\#2}$. Together with data from Supplementary Fig. 12a, they are used for quantitative calculation in Supplementary Fig. 12a. **b** Other two repeated pull-down assays to compare Brf2-binding capacity between mSNAPc$^{\#2}$ and mSNAPc$^{\#10}$. Together with data from Fig. 5e, they are used for quantitative calculation in Fig. 5f.

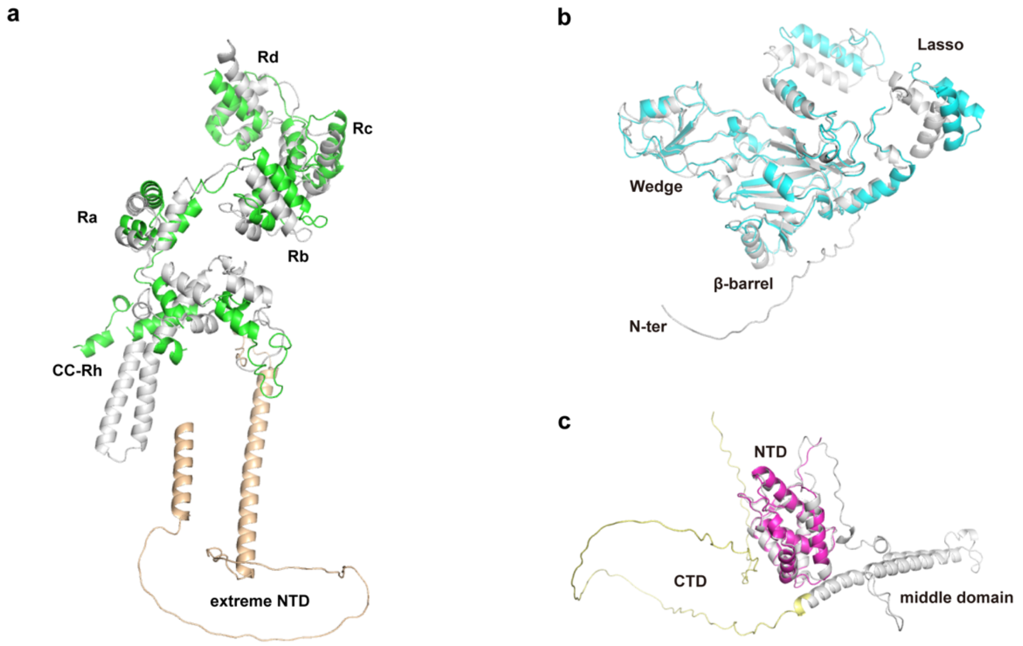

Supplementary Fig. 14 | The single-subunit superimpositions of experimental structures with predicted models by AlphaFold. a The NTD of SNAP190 (AlphaFold ID: AF-Q5SXM2-F1-model_v2). **b** SNAP50 (AlphaFold ID: AF-Q92966-F1-model_v2). **c** SNAP43 (AlphaFold ID: AF-Q16533-F1-model_v2). All predicted models are colored in silver, except the extreme NTD of SNAP190 and the CTD of SNAP43 are shown in wheat and yellow, separately.

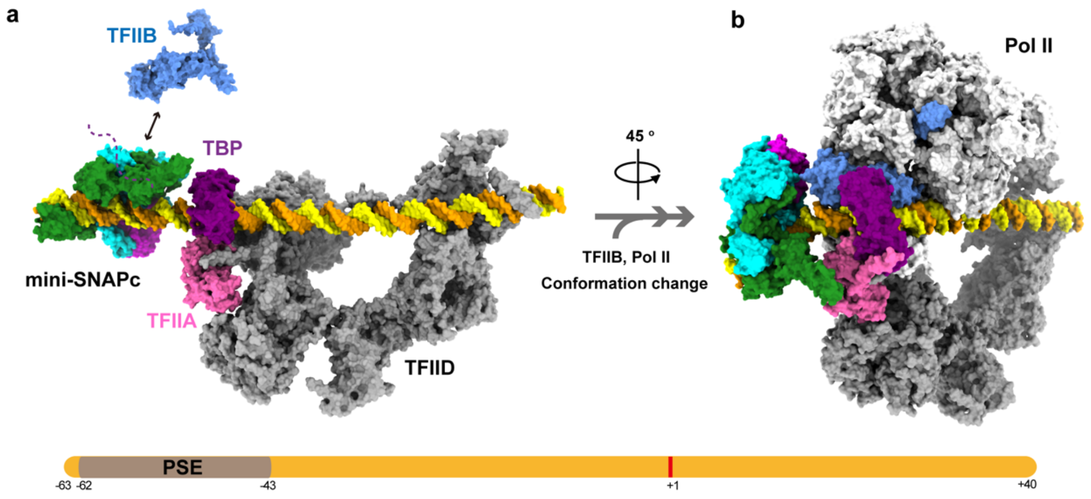

Supplementary Fig. 15 | The model of Pol II-dependent PIC assembled on TATA-less snRNA promoters. a A model of CP-TFIID^{ITL} in complex with mini-SNAPc and hU1 promoter, simulated by combining pre-PIC, namely CP^{SCP}-TFIID^{ITL} (PDB 7EGD) with our structure. In this model, the original bound DNA parts of mini-SNAPc and TFIIA-TFIID structures are substituted by hU1 promoter and coding-region (the bottom panel). In this stage, TBP functions as a part of TFIID, without bending dsDNA. The role of TFIIB remains unclear. **b** A model of core PIC containing mini-SNAPc, TFIIA, TBP-TFIID, TFIIB, TFIIF, and Pol II on hU1 promoter. The reference model is core-PIC on *puma* promoter (PDB 7EG8). This complex is formed after DNA-bending and module repositioning in a similar manner to canonical TATA-less mRNA promoters. In this stage, the CC-Rh domain of SNAP190 probably comes in contact with TFIIA. However, this interaction remains to be verified by further investigations.

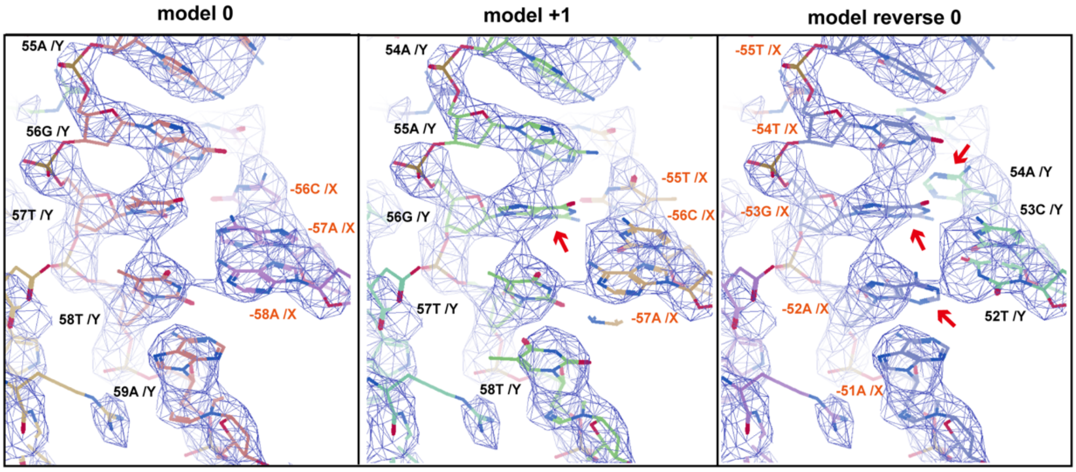

Supplementary Fig. 16 | The base sizes of pyrimidine and purine were used to evaluate PSE assignment. Under the current resolution, the density map of phosphate group of dsDNA can be positioned easily. Firstly, the positions of the phosphate skeleton of dsDNA were confirmed. As the sizes of base density are different, pyrimidine (T or C) and purine (A or G) can be distinguished clearly. Fourteen different atom models were built into the dsDNA map. The dsDNA model in the current structure is named “model 0”. Based on it, “model +1”, “model +2”, “model +3”, “model -1”, “model -2”, and “model -3” were generated by shifting upstream or downstream of 1, 2 or 3 bases, respectively. The other seven models come from the aforementioned models, reversed from 5’ to 3’ end, namely “model reverse 0”, “model reverse +1”, “model reverse +2”, “model reverse +3”, “model reverse -1”, “model reverse -2”, and “model reverse -3”. By evaluating the density map sizes of bases, “model 0” is confirmed to be the only correct model. Here, “model +1” and “model reverse 0” were listed to be compared to the correct “model 0”. The “model 0” of PSE is well-fitted into the density map. In “model +1”, the CG pair at position -56 (red arrow) does not match the electron density. In “model reverse 0”, the TA, GC, and AT pairs at positions -54 ~ -52 (red arrows) are beyond the scope of density map.

Supplementary Table 1 | The partial complexes of human SNAPc used in this study. The sample of mSNAPc#2 highlighted in grey was applied to cyro-EM study.

Name	SNAP190	SNAP50	SNAP43	SNAP19
mini-SNAPc	1-505	full-length	full-length	-
mSNAPc#1	1-505	full-length	full-length	full-length
mSNAPc#2	1-505	full-length	1-268	full-length
mSNAPc#3	140-505	full-length	1-150	-
mSNAPc#4	180-505	full-length	1-150	-
mSNAPc#5	(140-296)-4GS-(345-505)	full-length	1-150	-
mSNAPc#6	(180-296)-4GS-(345-505)	full-length	1-150	-
mSNAPc#7	140-505	1-351	1-150	-
mSNAPc#8	180-505	1-351	1-150	-
mSNAPc#9	140-505	141-411	1-150	-
mSNAPc#10	(1-182)-(273-505)	full-length	1-268	full-length
mSNAPc#11	1-143	-	148-268	full-length

Supplementary Table 2 | Cryo-EM data collection and refinement statistics.

**Data collection and processing**	
Microscope	Titan Krios G3i
Detector	Gatan K2 camera
Magnification	130,000
Voltage (kV)	300
Electron exposure (e^-/Å^2)	60
Defocus range (μM)	-1.0 to -2.5
Pixel size (Å/px)	0.538
Symmetry imposed	C1
Number of movies	4,937
Initial particle images	650,462
Final particle images	67,058
Map resolution (Å)	3.49
FSC threshold	0.143
**Model building and refinement**	
Overall correlation coefficients	
CC (mask)	0.77
CC (box)	0.77
CC (volume)	0.76
CC (peaks)	0.67
CC (main chain)	0.77
CC (side chain)	0.77
Model composition	
Chains	5
Residues	Protein: 814; Nucleotide: 48
Ligands	Zn: 2
RMSDs from ideal	
Bond length (Å^2)	0.005
Bond angle (^{o})	0.707
Validation	
Clash score	10.26
Rotamer outliers (%)	0

Ramachandran plot	
Favored (%)	91.21
Allowed (%)	8.79
Outlier (%)	0
