# Supplementary material for: Structural basis of human SNAPc recognizing proximal sequence element of snRNA promoter
Source: Nat Commun. 2022 Nov 11;13:6871. doi: 10.1038/s41467-022-34639-1 (PMC9652321; doi:10.1038/s41467-022-34639-1)
Supplement: Supplementary file 2 — Reporting Summary [file 41467_2022_34639_MOESM2_ESM.pdf]

nature portfolio

Corresponding author(s): Jianfeng Sun, Robert G. Roeder, Wei Wang

Last updated by author(s): Aug 30, 2022

Reporting Summary

Nature Portfolio wishes to improve the reproducibility of the work that we publish. This form provides structure for consistency and transparency in reporting. For further information on Nature Portfolio policies, see our Editorial Policies and the Editorial Policy Checklist.

Statistics

For all statistical analyses, confirm that the following items are present in the figure legend, table legend, main text, or Methods section.

n/a	Confirmed	
☐	☒	The exact sample size (*n*) for each experimental group/condition, given as a discrete number and unit of measurement
☐	☒	A statement on whether measurements were taken from distinct samples or whether the same sample was measured repeatedly
☒	☐	The statistical test(s) used AND whether they are one- or two-sided<br>*Only common tests should be described solely by name; describe more complex techniques in the Methods section.*
☒	☐	A description of all covariates tested
☒	☐	A description of any assumptions or corrections, such as tests of normality and adjustment for multiple comparisons
☐	☒	A full description of the statistical parameters including central tendency (e.g. means) or other basic estimates (e.g. regression coefficient) AND variation (e.g. standard deviation) or associated estimates of uncertainty (e.g. confidence intervals)
☒	☐	For null hypothesis testing, the test statistic (e.g. *F*, *t*, *r*) with confidence intervals, effect sizes, degrees of freedom and *P* value noted<br>*Give P values as exact values whenever suitable.*
☒	☐	For Bayesian analysis, information on the choice of priors and Markov chain Monte Carlo settings
☒	☐	For hierarchical and complex designs, identification of the appropriate level for tests and full reporting of outcomes
☒	☐	Estimates of effect sizes (e.g. Cohen's *d*, Pearson's *r*), indicating how they were calculated

Our web collection on statistics for biologists contains articles on many of the points above.

Software and code

Policy information about availability of computer code

Data collection	SerialEM 3.8
Data analysis	MotionCor2 1.4.0, CTFFIND4 4.1.8, cryoSPARC 3.2.0, Relion 3.1.1, Chimera 1.15, Coot 0.8.9.1, Pymol 1.7.4.2, Phenix 1.18.2, ChimeraX 1.3, AlphaFold v2.1.0, Amber 14.0, AmberTools 15.0.

For manuscripts utilizing custom algorithms or software that are central to the research but not yet described in published literature, software must be made available to editors and reviewers. We strongly encourage code deposition in a community repository (e.g. GitHub). See the Nature Portfolio guidelines for submitting code & software for further information.

Data

Policy information about availability of data

All manuscripts must include a data availability statement. This statement should provide the following information, where applicable:

- Accession codes, unique identifiers, or web links for publicly available datasets
- A description of any restrictions on data availability
- For clinical datasets or third party data, please ensure that the statement adheres to our policy

The atomic coordinates have been deposited in the Protein Data Bank (http://www.rcsb.org) with the access code 7XUR, and the EM maps have been deposited in EMDB (https://www.ebi.ac.uk/pdbe/emdb/) with the accession code EMD-33477. All data in this study are included in this Article and its Supplementary Information. Source data are also provided with this paper.

Human research participants

Policy information about studies involving human research participants and Sex and Gender in Research.

Reporting on sex and gender	Not applicable
Population characteristics	Not applicable
Recruitment	Not applicable
Ethics oversight	Not applicable

Note that full information on the approval of the study protocol must also be provided in the manuscript.

Field-specific reporting

Please select the one below that is the best fit for your research. If you are not sure, read the appropriate sections before making your selection.

- [x] Life sciences
- [ ] Behavioural & social sciences
- [ ] Ecological, evolutionary & environmental sciences

For a reference copy of the document with all sections, see nature.com/documents/nr-reporting-summary-flat.pdf

Life sciences study design

All studies must disclose on these points even when the disclosure is negative.

Sample size	Biochemical experiments were performed in biological triplicates (n=3) in order to allow for the calculation of mean values and the standard error of the mean.
Data exclusions	No data were excluded.
Replication	Biochemical experiments were repeated independently three times with similar results.
Randomization	No randomization was conducted as no decisions about inclusion or exclusion of data were taken and experiments were designed such that single parameters were varied during the experiment. All data was included in analysis.
Blinding	No blinding was conducted as no subjective decisions about data inclusion were involved in data collection.

Reporting for specific materials, systems and methods

We require information from authors about some types of materials, experimental systems and methods used in many studies. Here, indicate whether each material, system or method listed is relevant to your study. If you are not sure if a list item applies to your research, read the appropriate section before selecting a response.

Materials & experimental systems

n/a	Involved in the study	
[ ]	[x]	Antibodies
[ ]	[x]	Eukaryotic cell lines
[x]	[ ]	Palaeontology and archaeology
[x]	[ ]	Animals and other organisms
[x]	[ ]	Clinical data
[x]	[ ]	Dual use research of concern

Methods

n/a	Involved in the study	
[x]	[ ]	ChIP-seq
[x]	[ ]	Flow cytometry
[x]	[ ]	MRI-based neuroimaging

Antibodies

Antibodies used	Peimary antibodies: Mouse Monoclonal ANTI-FLAG® M2 antibody (Sigma-Aldrich, Cat#F3156, 1:400); Mouse Monoclonal StrepMAB-Classic HRP conjugate (IBA, Cat#2-1509-002, 1:10000); Rabbit TEV Cleavage Site Polyclonal Antibody (Thermo Fisher, Cat#PA1-119, 1:1000); Rabbit BRF2 Polyclonal antibody (Proteintech, Cat#12056-1-AP, 1:1000).<br>Secondary antibodies: Goat anti-rabbit IgG-HRP (Absin, Cat#abs20002, 1:10000); Goat anti-mouse IgG-HRP (Absin, Cat#abs20001, 1:10000).
Validation	1. Mouse Monoclonal ANTI-FLAG M2 antibody (Sigma-Aldrich, F3156) was validated by manufacturer to detect Flag-tagged fusion

proteins (https://www.sigmaaldrich.cn/CN/zh/product/sigma/f3165).
2. Mouse Monoclonal StrepMAB-Classic HRP conjugate (IBA, 2-1509-002) was validated by manufacturer to detect Strep-tag fusion proteins (https://www.iba-lifesciences.com/strepmab-classic-hrp-conjugate/2-1509-001).
3. Rabbit TEV Cleavage Site Polyclonal Antibody (Thermo Fisher, PA1-119) was validated by manufacturer to detect TEV cleavage site in fusion proteins (https://www.thermofisher.cn/cn/zh/antibody/product/TEV-Cleavage-Site-Antibody-Polyclonal/PA1-119).
4. Rabbit BRF2 Polyclonal antibody (Proteintech, 12056-1-AP,) was validated by manufacturer to detect Brf2 protein (https://www.ptgcn.com/products/BRF2-Antibody-12056-1-AP.htm)
5. Secondary antibodies were validated by manufacturer following these links. (https://www.absin.cn/goat-rabbit-igg-hrp/abs20002.html; https://www.absin.cn/goat-mouse-igg-hrp/abs20001.html)

Eukaryotic cell lines

Policy information about cell lines and Sex and Gender in Research

Cell line source(s)	The sf9 insect cells (Novagen) were used for protein expression in this study.
Authentication	The cell was routinely maintained in our laboratory. The cell was not authenticated.
Mycoplasma contamination	The cell line was test negative for mycoplasma contamination.
Commonly misidentified lines (See ICLAC register)	No commonly misidentified cell lines were used.
